# The Combination of Panobinostat and Melphalan for the Treatment of Patients with Multiple Myeloma

**DOI:** 10.3390/ijms232415671

**Published:** 2022-12-10

**Authors:** Maria Gkotzamanidou, Evangelos Terpos, Meletios A. Dimopoulos, Vassilis L. Souliotis

**Affiliations:** 1 Oncology Department, 251 Hellenic Air-Force General Hospital, 155 61 Athens, Greece; 2 Department of Clinical Therapeutics, School of Medicine, National and Kapodistrian University of Athens, 115 28 Athens, Greece; 3 Institute of Chemical Biology, National Hellenic Research Foundation, 116 35 Athens, Greece

**Keywords:** panobinostat, melphalan, multiple myeloma, DNA damage response, clinical response, combination therapy

## Abstract

Histone deacetylase inhibitors show synergy with several genotoxic drugs. Herein, we investigated the biological impact of the combined treatment of panobinostat and melphalan in multiple myeloma (MM). DNA damage response (DDR) parameters and the expression of DDR-associated genes were analyzed in bone marrow plasma cells (BMPCs) and peripheral blood mononuclear cells (PBMCs) from 26 newly diagnosed MM patients. PBMCs from 25 healthy controls (HC) were examined in parallel. Compared with the ex vivo melphalan-only treatment, combined treatment with panobinostat and melphalan significantly reduced the efficiency of nucleotide excision repair (NER) and double-strand-break repair (DSB/R), enhanced the accumulation of DNA lesions (monoadducts and DSBs), and increased the apoptosis rate only in patients’ BMPCs (all *p* < 0.001); marginal changes were observed in PBMCs from the same patients or HC. Accordingly, panobinostat pre-treatment decreased the expression levels of critical NER (DDB2, XPC) and DSB/R (MRE11A, PRKDC/DNAPKc, RAD50, XRCC6/Ku70) genes only in patients’ BMPCs; no significant changes were observed in PBMCs from patients or HC. Together, our findings demonstrate that panobinostat significantly increased the melphalan sensitivity of malignant BMPCs without increasing the melphalan sensitivity of PBMCs from the same patients, thus paving the way for combination therapies in MM with improved anti-myeloma efficacy and lower side effects.

## 1. Introduction

Multiple myeloma (MM), the second most common hematologic malignancy, is a plasma cell disorder characterized by the overproduction of monoclonal immunoglobulins [1]. High-dose melphalan followed by autologous stem-cell transplantation remains the backbone of the frontline treatment of MM patients [2,3,4]. Melphalan is nitrogen mustard, which is used in the treatment of several cancers [5]. It reacts with DNA and produces mostly N-alkylpurine-monoadducts, with a small fraction of them forming the extremely cytotoxic interstrand cross-links (ICLs) [6]. N-alkylpurine-monoadducts are repaired by the nucleotide excision repair (NER) mechanism, whereas homologous recombination (HR), NER, and translesion synthesis are required for the repair of ICL lesions [7,8,9]. Of note, ICL repair proceeds through the formation of double-strand breaks (DSBs), the most dangerous forms of DNA damage [10,11]. Despite the enormous advances in the treatment of MM, the disease is still incurable due to the development of resistance [12,13,14]. These findings point to a need for further research on new drugs and/or novel combinations of drugs with improved safety and clinical outcomes.

The latest research on therapies for MM has focused on histone deacetylases, enzymes that catalyze the removal of acetyl groups from the lysine residues of both histone and nonhistone proteins. Histone deacetylases are important gene expression regulators that act as transcriptional repressors and are often deregulated in several disorders including MM [15]. These properties have led to the use of histone deacetylase inhibitors (HDACi) to augment histone acetylation levels and ultimately induce cell cycle arrest and apoptosis in cancer cells [16]. A critical HDACi, namely panobinostat, was approved by the United States Food and Drug Administration (FDA) in February 2015 and the European Commission in September 2015, based on a Phase III subgroup analysis of its use as a combination therapy for relapsed/refractory MM patients who had received at least two prior lines of therapy [17]. Panobinostat is a pan-inhibitor of Class I, II, and IV histone deacetylases that inhibits the de-acetylation of both histone and nonhistone proteins, targeting lysine groups on chromatin, transcription factors, and several critical proteins including p53, heat shock protein-90, tubulin, and retinoblastoma protein [18]. This HDACi has shown antiproliferative and cytotoxic activities on cell lines and primary tumor cells from MM patients, refractory to several anti-MM drugs such as melphalan, doxorubicin, anthracycline, mitoxantrone, bortezomib, and dexamethasone [19,20,21]. Interestingly, using MM cell lines and an MM xenograft murine model, the combined treatment of panobinostat and melphalan showed a greater anti-myeloma effect than either panobinostat or melphalan alone [22]. Previous studies have also shown that the combined treatment of panobinostat and melphalan may be effective for patients with MM but at the cost of considerable toxicity [23,24,25,26,27].

To provide a better rationale for the selection of new drug combinations in MM, herein we investigated the mechanistic basis for the co-effect of panobinostat and melphalan and their characteristics. For this purpose, in bone marrow plasma cells (BMPCs) and peripheral blood mononuclear cells (PBMCs) from newly diagnosed MM patients, we evaluated the effect of this ex vivo combined treatment on critical DDR pathways including fundamental DNA repair mechanisms (NER, ICL repair, DSB repair) and apoptosis rates.

## 2. Results

### 2.1. Pre-Treatment with Panobinostat Significantly Increased Melphalan Sensitivity of BMPCs

The effect of the combined treatment of BMPCs with panobinostat and melphalan on critical DDR parameters, including DNA repair mechanisms (NER, ICL repair, DSB repair) and apoptosis rates, was evaluated in BMPCs from MM patients at baseline (16 responders and 9 non-responders to subsequent melphalan therapy; Table 1). For this purpose, BMPCs were pre-treated ex vivo with 5 nM panobinostat for 24 h, exposed to 100 μg/mL melphalan for 5 min in the presence of the HDACi, and the DDR parameters were followed for up to 48 h.

**Figure 1:**
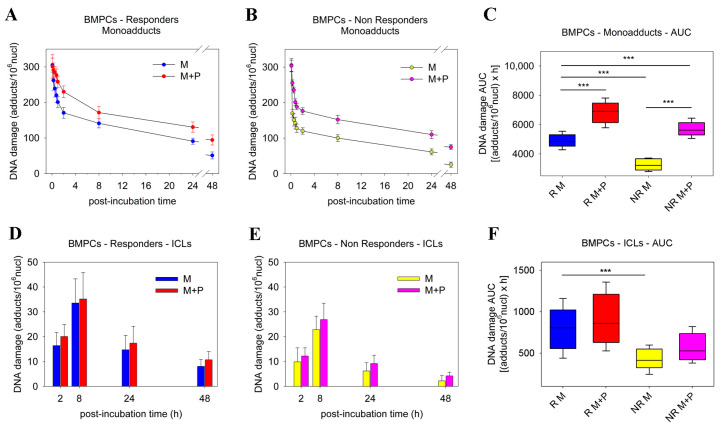
Kinetics of monoadduct and ICL formation and repair in BMPCs. The kinetics of monoadduct repair 0–48 h after treatment of BMPCs from (**A**) responder or (**B**) non-responder patients with melphalan ± panobinostat. (**C**) Accumulation of DNA damage (expressed as AUC) following treatment with melphalan ± panobinostat. The formation and repair of ICLs 2–48 h after treatment of BMPCs from (**D**) responder or (**E**) non-responder patients with melphalan ± panobinostat. (**F**) Accumulation of ICLs after treatment with melphalan ± panobinostat. The experiments shown were based on a minimum of three independent repeats. *** *p* < 0.001.

First, the efficiency of NER was analyzed at the active N-ras gene, the repair rate of which represents the total NER capacity [28]. In accordance with our previous data [29], following the 5 min melphalan treatment, a similar formation of monoadducts was observed in all MM patients examined, regardless of pre-treatment with panobinostat (Figure 1A,B). Then, a two-phase repair of melphalan-induced DNA damage was observed, with a fast component extending up to 2 h following melphalan treatment, and a slower progression of repair thereafter. Following melphalan-only treatment, responders’ BMPCs showed a slower monoadduct repair capacity than non-responders’ cells, resulting in a higher monoadduct burden (expressed as the area under the curve (AUC)] in responders’ cells (*p* < 0.001; Figure S1A,B). Notably, in both groups of MM patients, combined treatment with panobinostat and melphalan caused a significant reduction in the NER capacity and augmented the monoadduct burden compared with melphalan-only treatment (all *p* < 0.001; Figure 1C). No significant induction of monoadducts was observed following the panobinostat-only treatment of BMPCs.

Next, the kinetics of ICL formation and removal were examined. Regardless of pre-treatment with panobinostat, all patients showed maximal levels of ICLs within 8 h of melphalan treatment, which decreased thereafter (Figure 1D,E). In line with the monoadduct results, after melphalan-only treatment, responders showed higher ICL levels than non-responders (*p* < 0.001; Figure S1C,D). Interestingly, in both groups of MM patients, combined treatment with panobinostat and melphalan resulted in higher ICL levels at all time points analyzed and increased the ICL burden compared with melphalan-only treatment (Figure 1F); however, these results did not reach statistical significance. No significant induction of ICLs was found following the panobinostat-only treatment of BMPCs.

**Figure 2:**
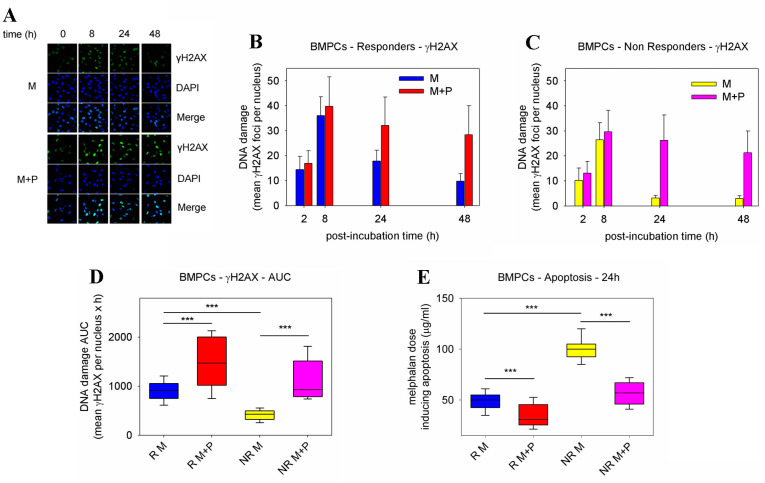
Formation and removal of γH2AX foci and induction of apoptosis in BMPCs. (**A**) Typical images showing the γH2AX staining at different time points after the ex vivo treatment of BMPCs from a representative non-responder patient with melphalan ± panobinostat. Upper images, immunofluorescence antigen staining; middle, cell nuclei labeled with DAPI; bottom, merged. M, melphalan; P, panobinostat. The formation and removal of γH2AX foci after treatment of BMPCs from (**B**) responder or (**C**) non-responder patients with melphalan ± panobinostat. (**D**) Accumulation of γH2AX foci expressed as AUC after treatment with melphalan ± panobinostat. (**E**) The induction of apoptosis 24 h after the ex vivo treatment of responder or non-responder patients with melphalan ± panobinostat. The experiments shown were based on a minimum of three independent repeats. *** *p* < 0.001.

To study the formation and repair of DSBs, γH2AX foci were measured using confocal microscopy (Figure 2A). Regardless of pre-treatment with panobinostat, all patients showed peak γH2AX foci levels within 8 h, which decreased thereafter (Figure 2B,C). Moreover, after melphalan-only treatment, responders’ BMPCs showed a lower γH2AX foci removal capacity and a significantly higher accumulation of γH2AX foci than non-responders (*p* < 0.001; Figure S1E,F). In both groups of patients, the combined treatment of panobinostat and melphalan resulted in the inhibition of γH2AX foci removal and significantly higher accumulation of γH2AX foci compared with melphalan-only treatment (*p* < 0.001; Figure 2D). No significant induction of γH2AX foci was observed after the panobinostat-only treatment of BMPCs.

The induction of apoptosis in BMPCs was measured at 24 h and 72 h following combined treatment with panobinostat and melphalan. Similar results were found at both time points analyzed, that is, in line with the DNA repair results, after melphalan-only treatment, BMPCs derived from responders showed lower concentrations of melphalan required for the induction of apoptosis compared with non-responders (all *p* < 0.001; Figure 2E and Figure S2A). These results indicate higher apoptotic rates in responders’ cells than in non-responders’ cells. Interestingly, we found that in both groups of patients, pre-treatment with panobinostat resulted in higher apoptosis rates compared with melphalan-only treatment. In addition, using the ApoTox-Glo Triplex Assay, the pre-treatment of BMPCs with panobinostat resulted in a significant enhancement of melphalan cytotoxicity and apoptosis rates (caspase activity), as well as decreased viability, compared with melphalan-only treatment (all *p* < 0.05; Figures S3 and S4). No significant changes in the cell viability, cytotoxicity, and apoptosis rates were obtained after the panobinostat-only treatment of BMPCs.

**Figure 3:**
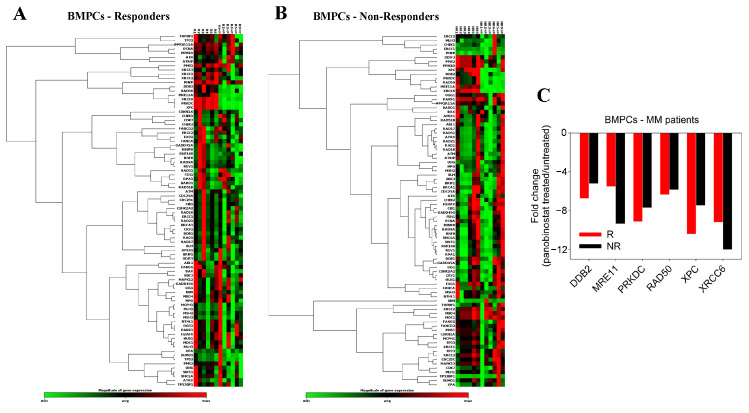
Panobinostat treatment on the expression of DDR-associated genes in BMPCs. Hierarchical clustergram of 84 DDR-associated genes in BMPCs from (**A**) 6 responder and (**B**) 6 non-responder patients. (**C**) Genes demonstrating at least a 2-fold difference in the transcription activity between panobinostat-treated and untreated MM patients. Gene acronyms are explained in Table S1.

The effect of panobinostat treatment on the expression of 84 DDR-associated genes was also examined in BMPCs from 12 MM patients (6 responders and 6 non-responders to subsequent melphalan therapy) (Figure 3A,B). In both groups of patients, we found that the ex vivo treatment of BMPCs with panobinostat for 24 h resulted in a marked decrease in the expression of critical NER-related genes, including DDB2 (damage-specific DNA binding protein 2) and XPC (Xeroderma pigmentosum, complementation group C), as well as the DSB repair genes MRE11A (meiotic recombination 11 homolog A), PRKDC/DNAPKc (DNA-dependent protein kinase catalytic subunit), RAD50, and XRCC6/Ku70 (X-ray repair cross-complementing 6) (Figure 3C and Figures S5–S7 and Table S2).

### 2.2. DDR Signals following Combined Treatment of PBMCs with Panobinostat and Melphalan

**Figure 4:**
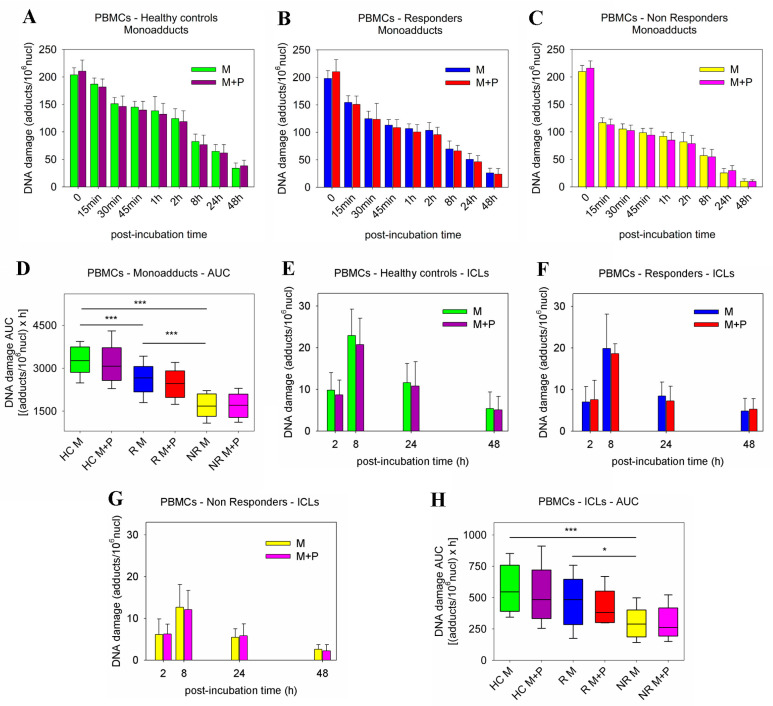
Kinetics of monoadduct repair in PBMCs. The kinetics of monoadduct repair 0–48 h after the ex vivo treatment of PBMCs from (**A**) healthy controls, (**B**) responder patients, and (**C**) non-responder patients with melphalan ± panobinostat. M, melphalan; P, panobinostat. (**D**) Accumulation of monoadducts (expressed as AUC) following treatment with melphalan ± panobinostat. The kinetics of ICL formation and repair 2–48 h after the ex vivo treatment of PBMCs from (**E**) healthy controls, (**F**) responder patients, and (**G**) non-responder patients with melphalan ± panobinostat. (**H**) Accumulation of ICLs expressed as AUC after treatment of PBMCs with melphalan ± panobinostat. The experiments shown were based on a minimum of three independent repeats. * *p* < 0.05, *** *p* < 0.001.

**Figure 5:**
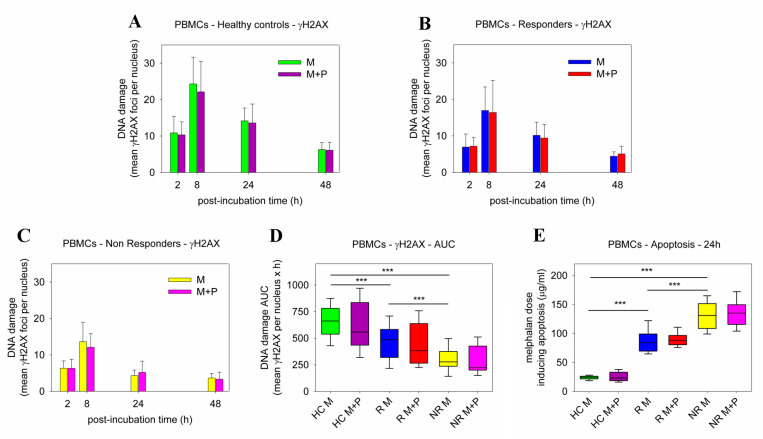
Formation/removal of γH2AX and induction of apoptosis in PBMCs. The formation and removal of γH2AX foci 2–48 h after the ex vivo treatment of PBMCs from (**A**) healthy controls, (**B**) responder patients, and (**C**) non-responder patients, with melphalan ± panobinostat. M, melphalan; P, panobinostat. (**D**) Accumulation of γH2AX foci expressed as AUC after treatment of PBMCs with melphalan ± panobinostat. (**E**) The induction of apoptosis 24 h after the ex vivo treatment of healthy controls and MM patients (responders or non-responders) with melphalan ± panobinostat. The experiments shown were based on a minimum of three independent repeats. *** *p* < 0.001.

Panobinostat-induced changes in DDR signals, including the efficiencies of NER, ICL repair, and DSB repair, as well as the apoptosis rates, were also evaluated in PBMCs from the 25 healthy controls and the 26 MM patients analyzed above. Following melphalan-only treatment, patients’ PBMCs showed higher repair capacities than HC (Figure S8A,C,E). Notably, PBMCs derived from non-responder patients showed slower NER, ICL/R, and DSB/R capacities than non-responders’ cells, resulting in a higher accumulation of DNA damage in responders’ cells (all *p* < 0.05; Figure S8B,D,E). More importantly, the co-treatment of PBMCs with panobinostat and melphalan resulted in marginal changes in the efficiencies of NER, ICL/R, and DSB/R (Figure 4A–C,E–G and Figure 5A–C); the DNA damage burden (Figure 4D,H and Figure 5D); and the apoptosis rates (Figure 5E and Figure S2B). In line with these results, using the ApoTox-Glo Triplex Assay, the ex vivo combined treatment with panobinostat and melphalan did not cause significant changes in the cell viability, cytotoxicity, and apoptosis rates of PBMCs (Figures S9–S11).

Next, the effect of panobinostat on the expression of DDR-associated genes was examined in PBMCs from the 6 HC and 12 newly diagnosed MM patients (6 responders and 6 non-responders) analyzed above. Contrary to the BMPC results, the treatment of PBMCs from HC and MM patients with panobinostat for 24 h did not significantly affect the expression levels of DDR genes (Figures S12–S15 and Table S3).

## 3. Discussion

Studies showing the interplay between the status of protein acetylation and apoptosis rates in MM cells suggest the use of the HDACi panobinostat as a therapeutic drug for MM. Interestingly, panobinostat has been shown to function synergistically with several chemical compounds including genotoxic drugs [20,21,22,23,24,25,26,27]. In this study, we investigate the molecular pathways targeted by the ex vivo combined treatment of panobinostat and melphalan and provide evidence that panobinostat enhances the melphalan sensitivity of BMPCs from MM patients, with marginal changes in the melphalan toxicity of PBMCs from the same patients.

It is known that the ability of MM cells to remove melphalan-induced DNA lesions represents a critical mechanism of resistance to melphalan therapy [29,30,31]. Therefore, in this study, using BMPCs from MM patients at baseline and responders and non-responders to subsequent melphalan therapy, we studied the main DNA repair mechanisms such as NER, ICL repair, and DSB repair. We found that, compared with melphalan-only treatment, the pre-treatment of BMPCs with panobinostat reduced the efficiencies of the NER and DSB repair mechanisms, resulting in the increased accumulation of DNA lesions (monoadducts and DSBs).

In order to explain these interesting results, the expression levels of critical DNA repair-associated genes were examined in both BMPCs and PBMCs following treatment with panobinostat. In BMPCs, we found that this HDACi acts as a modifier of the DNA repair machinery through the decrease in the expression of critical genes involved in DNA repair pathways, namely NER and DSB repair. The set of the decreased NER-related genes includes DDB2, which codes for the main factor involved in the recognition of UV-induced DNA lesions using the GGR (Global Genome Repair) subpathway of NER [32], and XPC, which codes for a NER-related protein that constantly scans the genome DNA in order to find DNA lesions [33]. As for the DSB-related genes, a significant decrease was found in the expression of the MRE11A gene, which codes for a nuclear protein with nuclease and intrinsic DNA binding activity, involved in the removal of DSBs and the maintenance of telomere length [34]; the PRKDC gene encoding the catalytic subunit of a DNA-PK, which is implicated in the non-homologous end-joining (NHEJ) subpathway of DSB repair and V(D)J recombination [35]; the RAD50 gene, which codes for a factor, which together with NBN (nibrin) and MRE11A (MRN complex), functions in the sensing and processing of DSBs, the S-phase checkpoint, the recombination of DNA, and the maintenance of telomeres [36]; and the XRCC6/Ku70 gene encoding for a molecular component of the NHEJ mechanism [37].

In line with these results, other studies have shown that HDAC inhibitors are indeed involved in the downregulation of DNA repair factors. Indeed, previous reports have shown that the HDACi vorinostat (suberoylanilide hydroxamic acid) downregulates the expression of the DSB repair proteins RAD50 and MRE11A in prostate and lung cancer cells [38], as well as the expression of the NHEJ proteins Ku70 and Ku80, and the homologous recombination factor RAD50 in melanoma cells [39]. Moreover, another HDACi, sodium butyrate, induced the downregulation of the NHEJ components Ku70, Ku80, and DNA-PK in human melanoma cell lines [40], whereas in acute myeloid leukemia cells, dacinostat (LAQ824) and entinostat (SNDX-275) downregulated BRCA1 (breast cancer type 1), RAD50, Ku80, EXO1 (exonuclease 1), and CHK2 (checkpoint kinase 2) [41].

In addition, we found that, compared with melphalan-only treatment, the combined treatment of BMPCs with panobinostat and melphalan significantly increased apoptosis rates. These results are in line with previous data showing that the treatment of prostate cancer cells with panobinostat in combination with radiation-induced increased apoptosis rates was comparable to radiation-only treatment [42]. In addition, MM cell lines showed induction of caspase-dependent apoptosis in response to panobinostat treatment; these effects were enhanced when panobinostat was combined with either melphalan or doxorubicin [22]. Moreover, in oral squamous cell carcinoma cell lines, vorinostat treatment increased tumor cell sensitivity to subtoxic doses of cisplatin [43]. Additionally, other studies have shown that inhibition of HDAC reduced the expression of survivin, a protein that prevents the induction of apoptosis by inhibiting the catalytic activity of caspases [44,45,46]. Other molecular mechanisms of HDACi lethality include free radical generation, interference with the chaperone protein function, and upregulation of the endogenous inhibitors of cell cycle progression [47].

On the other hand, in PBMCs from all groups of subjects examined, panobinostat pre-treatment induced only marginal changes in the repair efficiencies of melphalan-induced monoadducts (repaired by NER), ICLs, and DSBs; accumulation of DNA adducts; and apoptosis rates. In addition, the panobinostat pre-treatment of PBMCs did not significantly change the expression of critical DDR-related genes, thus explaining, at least in part, the selectivity of panobinostat in causing apoptosis in malignant BMPCs from MM patients at concentrations that caused little or no apoptotic cell death of PBMCs from the same patients. These results are in agreement with previous data showing that following treatment with vorinostat, normal but not transformed cells can readily repair DNA damage [38].

To conclude, our results show that panobinostat potentiates the melphalan sensitivity of malignant BMPCs without increasing the melphalan sensitivity of PBMCs from the same patients. Due to the fundamental reliance of MM upon DDR pathways, genotoxic drugs such as melphalan in combination with DDR modifiers such as panobinostat represent an exciting combinatorial therapeutic strategy, with improved anti-myeloma efficacy and possibly lower side effects.

## 4. Materials and Methods

### 4.1. Patients

PBMCs and BMPCs from twenty-six (*n* = 26) unselected newly diagnosed MM patients (12F/14M; median age 60 years, range 42–66) were studied (Table 1). Patients were staged according to the International Staging System (ISS) [48]. All patients received as first-line treatment HDM supported by ASCT. Response assessment was based on the International Myeloma Working Group (IMWG) criteria [49]. Patients were categorized according to their outcomes into responders (≥PR, *n* = 17) and non-responders (*n* = 9) to subsequent melphalan therapy. All patients’ samples were collected at diagnosis before treatment with any anti-myeloma or supportive treatment. Twenty-five (*n* = 25) healthy controls (HC; 12F/13M; median age 56 years, range 38–63) were analyzed in parallel.

Peripheral blood was collected from MM patients and healthy controls, and PBMCs were isolated as previously described [50]. Mononuclear cell suspensions were isolated from bone marrow aspirates by Ficoll gradient centrifugation (Ficoll-Paque Plus, Sigma Aldrich, St. Louis, MO, USA), and the isolation of plasma cells was carried out using CD138 microbeads and magnet-assisted cell sorting (MACS; Miltenyi Biotec, GmbH, Bergisch Gladbach, Germany). The purity of the plasma cells was evaluated using flow cytometry (Becton-Dickinson, San Jose, CA, USA) and was >90% in all samples analyzed.

### 4.2. Cell Treatment

Primary cells (PBMCs or BMPCs) were ex vivo treated with 100 μg/mL melphalan for 5 min at 37 °C in a complete RPMI-1640 medium supplemented with 10% fetal bovine serum, 100 units/mL penicillin, 100 mg/mL streptomycin, and 2 mmol/l L-glutamine. Cells were subsequently incubated in a drug-free medium for up to 48 h, harvested, and stored at −70 °C. In the combination experiments, cells were pretreated with 5 nM of panobinostat (LBH589; Selleck Chemicals, #S1030) for 24 h at 37 °C, treated with 100 μg/mL melphalan for 5 min in the presence of the HDACi, incubated in a drug-free medium for up to 48 h, harvested, and stored at −70 °C.

### 4.3. Measurement of Gene-Specific Damage Repair

Gene-specific N-alkylpurine-monoadducts and ICLs were measured in the N-ras gene using Southern blot analysis as described in [28]. Briefly, following treatment of primary cells (PBMCs and BMPCs) with melphalan for 5 min at 37 °C, and incubation of cells in a drug-free medium for up to 48 h, genomic DNA was isolated, digested with the restriction enzyme EcoRI, and heated at 70 °C for 30 min for depurination of N-alkylated bases. Then, the apurinic sites were converted to single-strand breaks by incubation with NaOH for 30 min at 37 °C and the DNA was size fractionated in agarose gel and Southern blotted. For the measurement of DNA interstrand cross-links, following the isolation of genomic DNA and restriction enzyme digestion as described above, the alkylations were not converted to strand breaks and DNA was denatured before agarose gel electrophoresis and Southern blotting. Hybridizations and calculation of the average frequency of melphalan-induced monoadducts and DNA interstrand cross-links were performed as described previously [28].

### 4.4. Measurement of γH2AX Foci

Aliquots of 2 × 10^4^ cells were adhered to the coverslip, fixed, and stored at −70 °C until the analysis of γH2AX foci using immunofluorescence antigen staining and confocal laser scanning microscope analysis [29]. Briefly, primary cells (PBMCs and BMPCs) were incubated with γH2AX phosphospecific antibody (Cell-Signaling Technology, #9718T, Danvers, MA, USA), washed, and incubated with fluorescent secondary antibody (Alexa Fluor 488 goat anti-mouse IgG; Abcam, Cambridge, UK) and images were visualized with a confocal laser scanning microscope (Leica TCS SP-1). Foci were manually counted in at least 200 cells/treatment conditions and the results were expressed as the mean γH2AX foci per nucleus from 3 independent experiments.

### 4.5. Apoptosis and Cell Viability Assay

Aliquots of 2 × 10^4^ cells were treated with doses of melphalan (0–200 μg/mL) for 5 min in the presence or not of 5 nM panobinostat, followed by a 24 h or 72 h post-incubation time. Apoptosis rates were measured using the Cell Death Detection ELISA-PLUS kit (Roche Applied Sciences, Penzberg, Germany) according to the manufacturer’s instructions. ApoTox-Glo Triplex Assay (Promega, Mannheim, Germany) was used to measure the cells’ viability, cytotoxicity, and apoptosis at 24 h or 72 h according to the manufacturer’s instructions.

### 4.6. Expression of DDR-Associated Genes

For the extraction of total RNA from freshly isolated primary cells (PBMCs and BMPCs), we used the RNeasy kit (Qiagen, #74104, Hilden, Germany) according to the manufacturer’s protocol. Then, to identify the differentially expressed genes, PCR array analysis using the RT² Profiler™ PCR-Array of 84 genes related to the DDR network (QIAGEN, #PAHS-029Z; Table S1) and data analysis using the RT^2^ Profiler PCR Array Data Analysis web portal (https://geneglobe.qiagen.com/gr/analyze/ (accessed on 3 August 2021) were performed as previously described [51].

### 4.7. Statistical Analysis

All data from DNA repair assessment for PBMCs and BMPCs are shown as mean values ± standard deviations (SD). Comparisons within PBMCs and BMPCs, between the combined panobinostat and melphalan therapy groups, and the melphalan-only groups were performed using the Wilcoxon rank-sum statistical test. The Student’s *t-*test was used to determine the differences in cell viability, cytotoxicity, and Caspase-3/7 activity. A *p*-value less than 0.05 was considered statistically significant.

## Figures and Tables

**Table 1:** Patient and disease characteristics.

Characteristic		Patient		
		No.	Age (years)	% of Total
Sex	Female	12		46.2
	Male	14		53.8
Age	Median		60	
	Range		42–66	
Ig subtype	IgG	12		46.2
	IgA	9		34.8
	IgM	0		0
	IgE	0		0
	FLCs	5		19.2
	Non-secretory	0		0
ISS stage	I	5		19.2
	II	8		30.8
	III	13		50.0
Response to HDM	Responders	17		65.4
	Non-Responders	9		34.6
High-risk cytogenetics *	Responders	6		35.3
	Non-Responders	4		44.4

* High-risk cytogenetics are defined as the presence of t (4; 14), t (4; 20), deletion 17p13, or 1q21 gain.

## Data Availability

Data are available upon reasonable request.

## Supplementary Materials

The supporting information can be downloaded at: https://www.mdpi.com/article/10.3390/ijms232415671/s1.
