# Supplementary material for: The Combination of Panobinostat and Melphalan for the Treatment of Patients with Multiple Myeloma
Source: Int J Mol Sci. 2022 Dec 10;23(24):15671. doi: 10.3390/ijms232415671 (PMC9778728; doi:10.3390/ijms232415671)
Supplement: Supplementary file 1 [file ijms-23-15671-s001.zip › ijms-2089594-supplementary.pdf]

Supplementary Materials

The combination of panobinostat and melphalan for the treatment of patients with multiple myeloma

Table of Contents

- Table S1. RT² Profiler™ PCR Array Human DNA Damage Signaling Pathway: Gene list
- Table S2. Genes under/over-expressed in BMPCs from panobinostat-treated *versus* untreated MM patients
- Table S3. Genes under/over-expressed in PBMCs from panobinostat-treated *versus* untreated MM patients
- Figure S1. Kinetics of melphalan-induced DNA damage formation and repair in BMPCs
- Figure S2. Apoptosis rates 72h after treatment
- Figure S3. ApoTox-Glo triplex assay of BMPCs from responders patients
- Figure S4. ApoTox-Glo triplex assay of BMPCs from non-responders patients
- Figure S5. Significant gene expression changes between panobinostat treated and non-treated BMPCs - Scatter Plot
- Figure S6. Significant gene expression changes between panobinostat treated and non-treated BMPCs - Volcano Plot
- Figure S7. Heat Map - BMPCs
- Figure S8. Kinetics of melphalan-induced DNA damage formation and repair in PBMCs
- Figure S9. ApoTox-Glo triplex assay of PBMCs from healthy controls
- Figure S10. ApoTox-Glo triplex assay of PBMCs from responders patients
- Figure S11. ApoTox-Glo triplex assay of PBMCs from non-responders patients
- Figure S12. Panobinostat treatment on the expression of DDR-associated genes in PBMCs - Hierarchical clustergrams
- Figure S13. Significant gene expression changes between panobinostat treated and non-treated PBMCs - Scatter Plot
- Figure S14. Significant gene expression changes between panobinostat treated and non-treated PBMCs - Volcano Plot
- Figure S15. Heat Map - PBMCs

Table S1. RT² Profiler™ PCR Array Human DNA Damage Signaling Pathway: Gene list

No	Symbol	GeneBank	Description
1	ABL1	NM_005157	C-abl oncogene 1, non-receptor tyrosine kinase
2	APEX1	NM_080649	APEX nuclease (multifunctional DNA repair enzyme) 1
3	ATM	NM_000051	Ataxia telangiectasia mutated
4	ATR	NM_001184	Ataxia telangiectasia and Rad3 related
5	ATRIP	NM_032166	ATR interacting protein
6	ATRX	NM_000489	Alpha thalassemia/mental retardation syndrome X-linked
7	BARD1	NM_000465	BRCA1 associated RING domain 1
8	BAX	NM_004324	BCL2-associated X protein
9	BBC3	NM_014417	BCL2 binding component 3
10	BLM	NM_000057	Bloom syndrome, RecQ helicase-like
11	BRCA1	NM_007294	Breast cancer 1, early onset
12	BRIP1	NM_032043	BRCA1 interacting protein C-terminal helicase 1
13	CDC25A	NM_001789	Cell division cycle 25 homolog A (S. pombe)
14	CDC25C	NM_001790	Cell division cycle 25 homolog C (S. pombe)
15	CDK7	NM_001799	Cyclin-dependent kinase 7
16	CDKN1A	NM_000389	Cyclin-dependent kinase inhibitor 1A (p21, Cip1)
17	CHEK1	NM_001274	CHK1 checkpoint homolog (S. pombe)
18	CHEK2	NM_007194	CHK2 checkpoint homolog (S. pombe)
19	CIB1	NM_006384	Calcium and integrin binding 1 (calmyrin)
20	CRY1	NM_004075	Cryptochrome 1 (photolyase-like)
21	CSNK2A2	NM_001896	Casein kinase 2, alpha prime polypeptide
22	DDB1	NM_001923	Damage-specific DNA binding protein 1, 127kDa
23	DDB2	NM_000107	Damage-specific DNA binding protein 2, 48kDa
24	DDIT3	NM_004083	DNA-damage-inducible transcript 3
25	ERCC1	NM_001983	Excision repair cross-complementing rodent repair deficiency, complementation group 1 (includes overlapping antisense sequence)
26	ERCC2	NM_000400	Excision repair cross-complementing rodent repair deficiency, complementation group 2
27	EXO1	NM_130398	Exonuclease 1
28	FANCA	NM_000135	Fanconi anemia, complementation group A
29	FANCD2	NM_033084	Fanconi anemia, complementation group D2
30	FANCG	NM_004629	Fanconi anemia, complementation group G
31	FEN1	NM_004111	Flap structure-specific endonuclease 1
32	GADD45A	NM_001924	Growth arrest and DNA-damage-inducible, alpha
33	GADD45G	NM_006705	Growth arrest and DNA-damage-inducible, gamma
34	H2AFX	NM_002105	H2A histone family, member X
35	HUS1	NM_004507	HUS1 checkpoint homolog (S. pombe)
36	LIG1	NM_000234	Ligase I, DNA, ATP-dependent
37	MAPK12	NM_002969	Mitogen-activated protein kinase 12
38	MBD4	NM_003925	Methyl-CpG binding domain protein 4
39	MCPH1	NM_024596	Microcephalin 1
40	MDC1	NM_014641	Mediator of DNA-damage checkpoint 1
41	MLH1	NM_000249	MutL homolog 1, colon cancer, nonpolyposis type 2 (E. coli)
42	MLH3	NM_014381	MutL homolog 3 (E. coli)
43	MPG	NM_002434	N-methylpurine-DNA glycosylase

44	MRE11A	NM_005590	MRE11 meiotic recombination 11 homolog A (S. cerevisiae)
45	MSH2	NM_000251	MutS homolog 2, colon cancer, nonpolyposis type 1 (E. coli)
46	MSH3	NM_002439	MutS homolog 3 (E. coli)
47	NBN	NM_002485	Nibrin
48	NTHL1	NM_002528	Nth endonuclease III-like 1 (E. coli)
49	OGG1	NM_002542	8-oxoguanine DNA glycosylase
50	PARP1	NM_001618	Poly (ADP-ribose) polymerase 1
51	PCNA	NM_182649	Proliferating cell nuclear antigen
52	PMS1	NM_000534	PMS1 postmeiotic segregation increased 1 (S. cerevisiae)
53	PMS2	NM_000535	PMS2 postmeiotic segregation increased 2 (S. cerevisiae)
54	PNKP	NM_007254	Polynucleotide kinase 3'-phosphatase
55	PPM1D	NM_003620	Protein phosphatase, Mg2+/Mn2+ dependent, 1D
56	PPP1R15A	NM_014330	Protein phosphatase 1, regulatory (inhibitor) subunit 15A
57	PRKDC	NM_006904	Protein kinase, DNA-activated, catalytic polypeptide
58	RAD1	NM_002853	RAD1 homolog (S. pombe)
59	RAD17	NM_002873	RAD17 homolog (S. pombe)
60	RAD18	NM_020165	RAD18 homolog (S. cerevisiae)
61	RAD21	NM_006265	RAD21 homolog (S. pombe)
62	RAD50	NM_005732	RAD50 homolog (S. cerevisiae)
63	RAD51	NM_002875	RAD51 homolog (S. cerevisiae)
64	RAD51B	NM_133509	RAD51 homolog B (S. cerevisiae)
65	RAD9A	NM_004584	RAD9 homolog A (S. pombe)
66	RBBP8	NM_002894	Retinoblastoma binding protein 8
67	REV1	NM_016316	REV1 homolog (S. cerevisiae)
68	RNF168	NM_152617	Ring finger protein 168
69	RNF8	NM_183078	Ring finger protein 8
70	RPA1	NM_002945	Replication protein A1, 70kDa
71	SIRT1	NM_012238	Sirtuin 1
72	SMC1A	NM_006306	Structural maintenance of chromosomes 1A
73	SUMO1	NM_003352	SMT3 suppressor of mif two 3 homolog 1 (S. cerevisiae)
74	TOPBP1	NM_007027	Topoisomerase (DNA) II binding protein 1
75	TP53	NM_000546	Tumor protein p53
76	TP53BP1	NM_005657	Tumor protein p53 binding protein 1
77	TP73	NM_005427	Tumor protein p73
78	UNG	NM_003362	Uracil-DNA glycosylase
79	XPA	NM_000380	Xeroderma pigmentosum, complementation group A
80	XPC	NM_004628	Xeroderma pigmentosum, complementation group C
81	XRCC1	NM_006297	X-ray repair complementing defective repair in Chinese hamster cells 1
82	XRCC2	NM_005431	X-ray repair complementing defective repair in Chinese hamster cells 2
83	XRCC3	NM_005432	X-ray repair complementing defective repair in Chinese hamster cells 3
84	XRCC6	NM_001469	X-ray repair complementing defective repair in Chinese hamster cells 6

Table S2. Genes under/over-expressed in BMPCs from panobinostat-treated *versus* untreated MM patients

No	Symbol	Up-Down Regulation (panobinostat treated/untreated)	
		Responders	Non Responders
1	ABL1	-1.09	-1.42
2	APEX1	-1.08	-1.14
3	ATM	-1.24	-1.00
4	ATR	-1.08	-1.24
5	ATRIP	-1.08	-1.19
6	ATRX	-1.13	-1.12
7	BARD1	-1.12	-1.09
8	BAX	-1.59	-1.09
9	BBC3	-1.59	-1.06
10	BLM	-1.14	1.10
11	BRCA1	-1.41	-1.39
12	BRIP1	-1.03	1.06
13	CDC25A	-1.26	1.04
14	CDC25C	-1.18	-1.02
15	CDK7	1.04	1.01
16	CDKN1A	-1.19	-1.25
17	CHEK1	1.01	-1.07
18	CHEK2	-1.16	-1.04
19	CIB1	-1.28	-1.01
20	CRY1	-1.29	-1.00
21	CSNK2A2	-1.23	-1.08
22	DDB1	-1.18	-1.14
23	DDB2	**-6.70**	**-5.18**
24	DDIT3	-1.02	-1.11
25	ERCC1	-1.28	-1.09
26	ERCC2	-1.34	-1.10
27	EXO1	-1.23	-1.12
28	FANCA	-1.37	-1.11
29	FANCD2	-1.05	-1.05
30	FANCG	-1.13	-1.10
31	FEN1	-1.11	-1.33
32	GADD45A	-1.27	-1.03
33	GADD45G	-1.02	-1.05
34	H2AFX	-1.16	-1.35
35	HUS1	-1.22	-1.06
36	LIG1	-1.06	1.05

37	MAPK12	-1.02	-1.08
38	MBD4	-1.13	-1.02
39	MCPH1	-1.13	-1.19
40	MDC1	-1.19	-1.03
41	MLH1	-1.10	-1.08
42	MLH3	-1.15	1.01
43	MPG	-1.21	-1.07
44	MRE11A	**-5.48**	**-9.31**
45	MSH2	-1.15	1.06
46	MSH3	-1.04	-1.03
47	NBN	-1.16	1.42
48	NTHL1	-1.23	-1.03
49	OGG1	-1.09	1.01
50	PARP1	-1.13	-1.19
51	PCNA	-1.16	-1.33
52	PMS1	-1.13	-1.08
53	PMS2	-1.50	-1.07
54	PNKP	-1.28	1.34
55	PPM1D	-1.18	-1.07
56	PPP1R15A	-1.38	-1.08
57	PRKDC	**-9.10**	**-7.66**
58	RAD1	-1.27	-1.10
59	RAD17	-1.21	-1.02
60	RAD18	-1.21	-1.13
61	RAD21	-1.32	-1.16
62	RAD50	**-6.31**	**-5.81**
63	RAD51	1.13	-1.12
64	RAD51B	-1.06	-1.11
65	RAD9A	-1.35	-1.06
66	RBBP8	-1.31	-1.17
67	REV1	-1.35	1.10
68	RNF168	-1.20	-1.01
69	RNF8	-1.23	-1.07
70	RPA1	-1.07	-1.04
71	SIRT1	-1.13	-1.00
72	SMC1A	-1.23	-1.10
73	SUMO1	-1.13	-1.05
74	TOPBP1	-1.15	1.12
75	TP53	-1.15	-1.11
76	TP53BP1	-1.09	1.28

77	TP73	-1.08	1.01
78	UNG	-1.27	-1.05
79	XPA	-1.08	1.11
80	XPC	**-10.37**	**-7.43**
81	XRCC1	-1.20	-1.03
82	XRCC2	-1.18	-1.08
83	XRCC3	-1.07	-1.06
84	XRCC6	**-9.17**	**-11.98**

Table S3. Genes under/over-expressed in PBMCs from panobinostat-treated *versus* untreated MM patients

No	Symbol	Up-Down Regulation (panobinostat treated/untreated)		
		Healthy controls	Responders	Non Responders
1	ABL1	-1.14	1.1	1.08
2	APEX1	-1.07	1.16	-1.02
3	ATM	1.04	1.42	1.02
4	ATR	1.01	1.24	-1
5	ATRIP	-1.13	1.13	1.48
6	ATRX	-1.06	1.2	-1.02
7	BARD1	-1	1.33	1.19
8	BAX	-1.11	1.22	1.06
9	BBC3	-1.03	1.26	1
10	BLM	-1.05	1.7	1
11	BRCA1	1.06	1.19	-1.08
12	BRIP1	-1.06	1.26	-1.06
13	CDC25A	-1.41	1.19	1.02
14	CDC25C	-1.08	1.13	-1.03
15	CDK7	-1.12	1.04	-1.04
16	CDKN1A	-1.12	1.22	1.02
17	CHEK1	-1.16	1.17	-1.12
18	CHEK2	-1.07	1.14	-1.11
19	CIB1	-1.05	1.12	-1.04
20	CRY1	-1.07	1.14	-1.02
21	CSNK2A2	-1.11	1.34	1.06
22	DDB1	-1.05	1.17	-1.12
23	DDB2	1.08	1.29	-1.03
24	DDIT3	1.12	1.04	-1.08
25	ERCC1	-1.17	1.17	1.05
26	ERCC2	-1.12	1.28	1.03

27	EXO1	-1.09	1.26	-1.1
28	FANCA	-1.01	1.26	-1.01
29	FANCD2	-1.04	1.14	-1.08
30	FANCG	1.05	1.06	1.13
31	FEN1	-1.08	1.25	1.17
32	GADD45A	-1.1	1.11	1.03
33	GADD45G	1.07	1.13	-1.03
34	H2AFX	1.05	1.21	-1.06
35	HUS1	-1.02	1.21	-1
36	LIG1	1	1.09	-1.13
37	MAPK12	1.29	1.21	1.23
38	MBD4	-1.14	1.08	1.12
39	MCPH1	-1.07	1.3	1.25
40	MDC1	1.17	1.13	1.03
41	MLH1	1.07	1.24	1.17
42	MLH3	-1.06	1.16	1.05
43	MPG	-1.28	1.07	-1.11
44	MRE11A	1.02	1.14	1.15
45	MSH2	-1.05	1.11	-1.06
46	MSH3	-1.17	1.18	-1.11
47	NBN	-1.06	1.28	1.09
48	NTHL1	-1.18	1.17	-1.13
49	OGG1	-1.49	1.14	-1.02
50	PARP1	-1.08	1.18	1.01
51	PCNA	1.28	1.1	1.04
52	PMS1	-1.01	1.15	1.06
53	PMS2	-1.02	1.06	1.1
54	PNKP	-1.11	1.15	1.1
55	PPM1D	1.01	1.23	1.05
56	PPP1R15A	1.02	1.17	-1.1
57	PRKDC	-1.12	1.12	-1.03
58	RAD1	-1.06	1.06	1.08
59	RAD17	1.03	1.23	1.08
60	RAD18	1.02	1.19	1.19
61	RAD21	-1.03	1.19	-1.06
62	RAD50	-1.05	1.17	1.14
63	RAD51	-1.04	1.11	1.15
64	RAD51B	1.01	1.12	-1.03
65	RAD9A	-1.12	1.19	1.11
66	RBBP8	-1.36	1.03	1.01

67	REV1	-1.13	1.2	-1.04
68	RNF168	-1.22	1.19	1.06
69	RNF8	-1.06	1.23	-1.04
70	RPA1	-1.06	1.16	1
71	SIRT1	-1.02	1.1	1.05
72	SMC1A	-1.34	1.16	-1
73	SUMO1	-1.13	1.19	-1.02
74	TOPBP1	-1	1.09	1.06
75	TP53	-1.14	1.18	-1
76	TP53BP1	-1.03	1.09	1.19
77	TP73	-1.02	1.18	1.2
78	UNG	1.06	1.17	-1.32
79	XPA	1.07	1.12	-1.01
80	XPC	-1.02	1.13	-1.04
81	XRCC1	-1.06	1.04	-1.01
82	XRCC2	1.31	1.11	-1.01
83	XRCC3	-1.05	1.22	-1.05
84	XRCC6	-1.12	-1.16	1.06

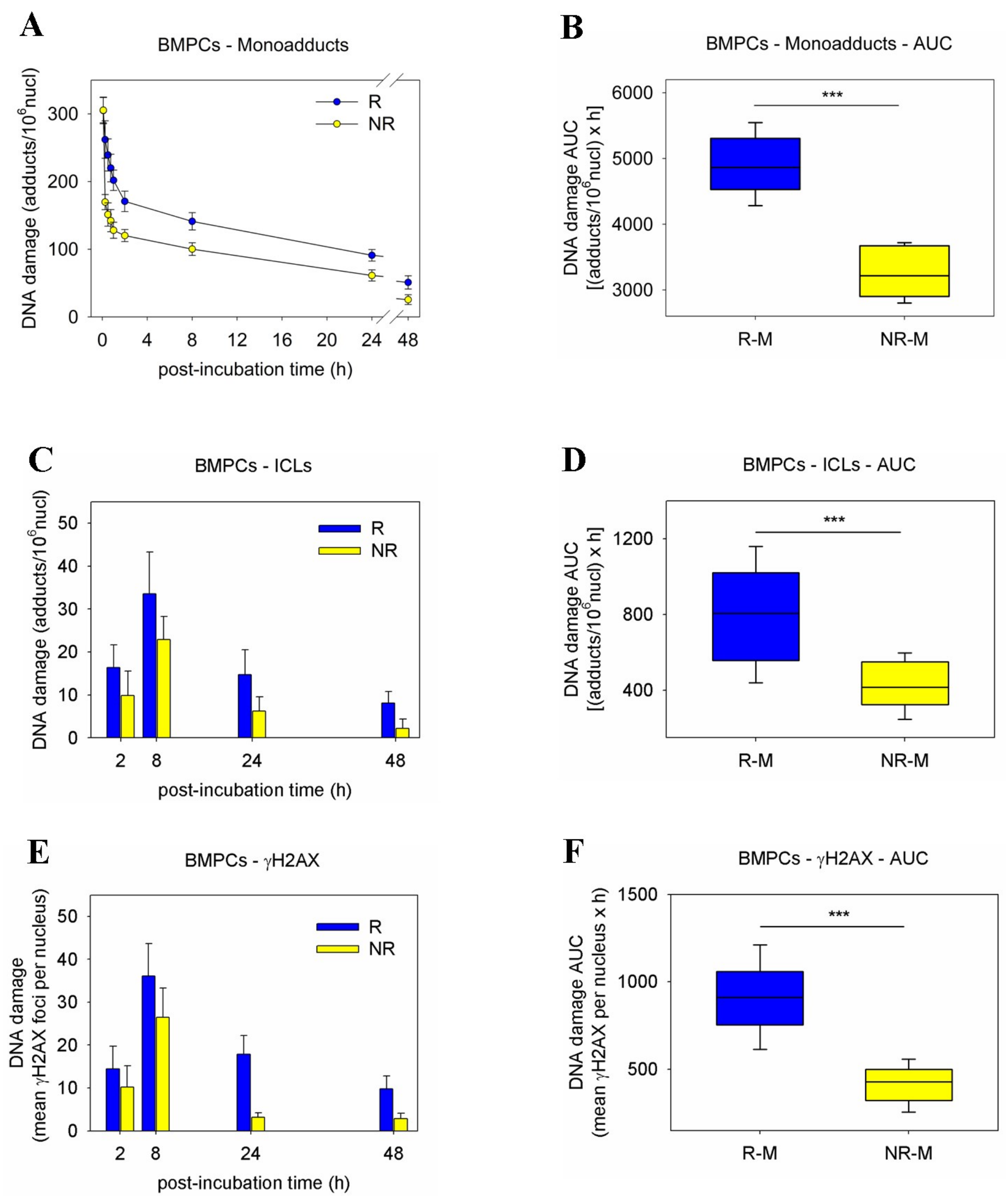

Figure S1. Kinetics of melphalan-induced DNA damage formation and repair in BMPCs. (**A**) The kinetics of monoadducts repair 0-48h after treatment of BMPCs from responders (R) or non-responders (NR) patients with melphalan (M). (**B**) Accumulation of DNA damage (expressed as AUC) following treatment with melphalan. The formation and repair of ICLs (**C**) and the accumulation of ICLs (**D**) after treatment with melphalan. The formation and removal of γH2AX foci (**E**) and the accumulation of γH2AX foci (**F**) after treatment with melphalan. The experiments shown were based on a minimum of three independent repeats; ***$p < 0.001$.

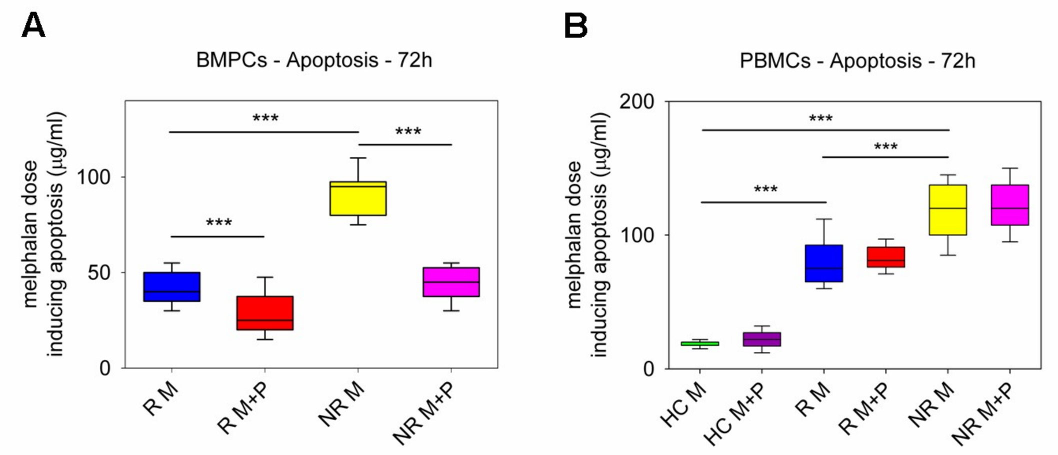

Figure S2. Apoptosis rates 72h after melphalan ± panobinostat treatment. The induction of apoptosis 72h after the *ex vivo* treatment of BMPCs (**A**) and PBMCs (**B**) with melphalan ± panobinostat. The experiments shown were based on a minimum of three independent repeats; ***$p < 0.001$.

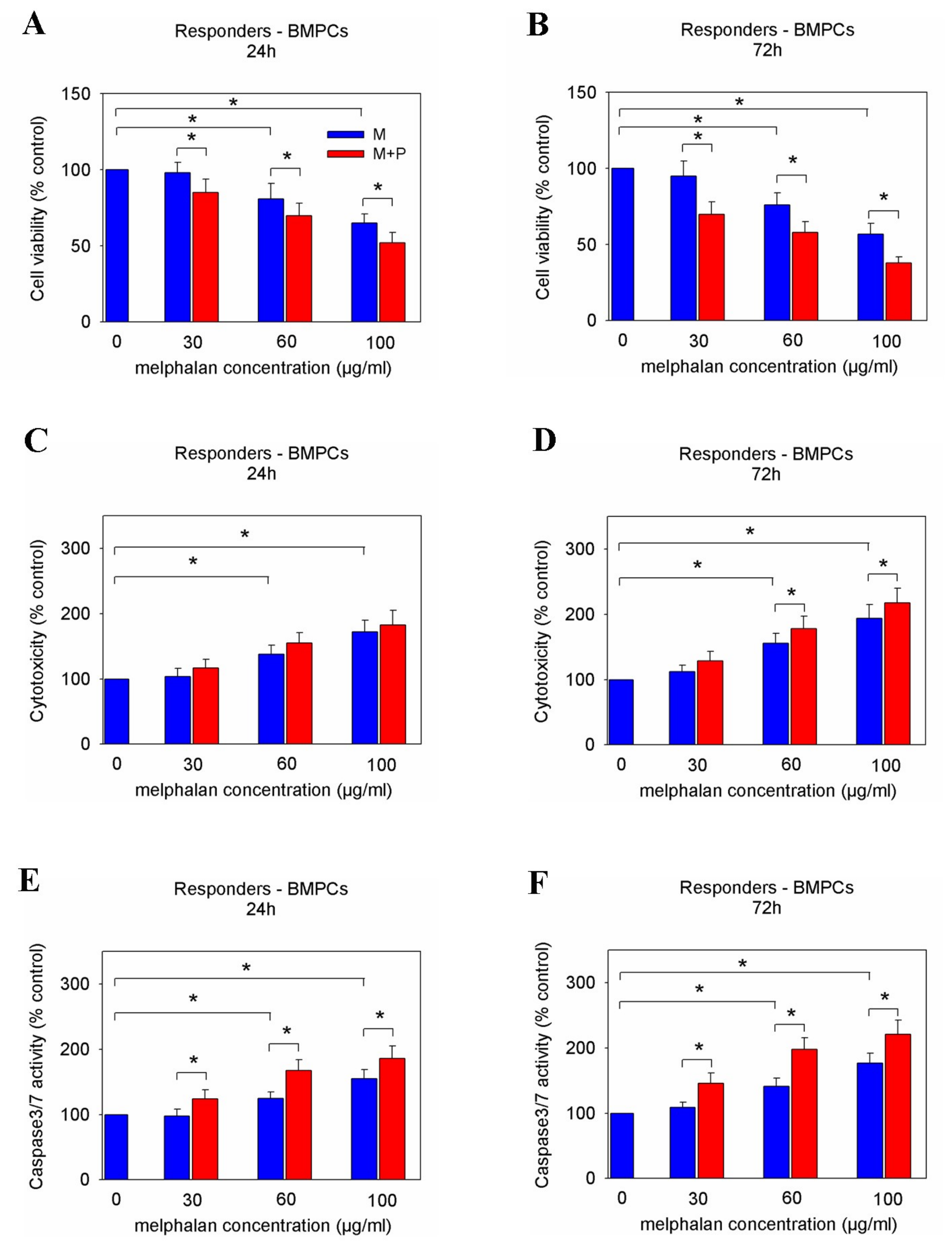

Figure S3. ApoTox-Glo triplex assay of BMPCs from responders patients. The viability (**A**,**B**), cytotoxicity (**C**,**D**), and apoptosis (**E**,**F**) of BMPCs from responders 24h (**A**,**C**,**E**) and 72h (**B**,**D**,**F**) after treatment with various doses of melphalan (0-100µg/ml) in combination or not of 5nM of panobinostat. Data are presented as the mean ± SD; *$p < 0.05$.

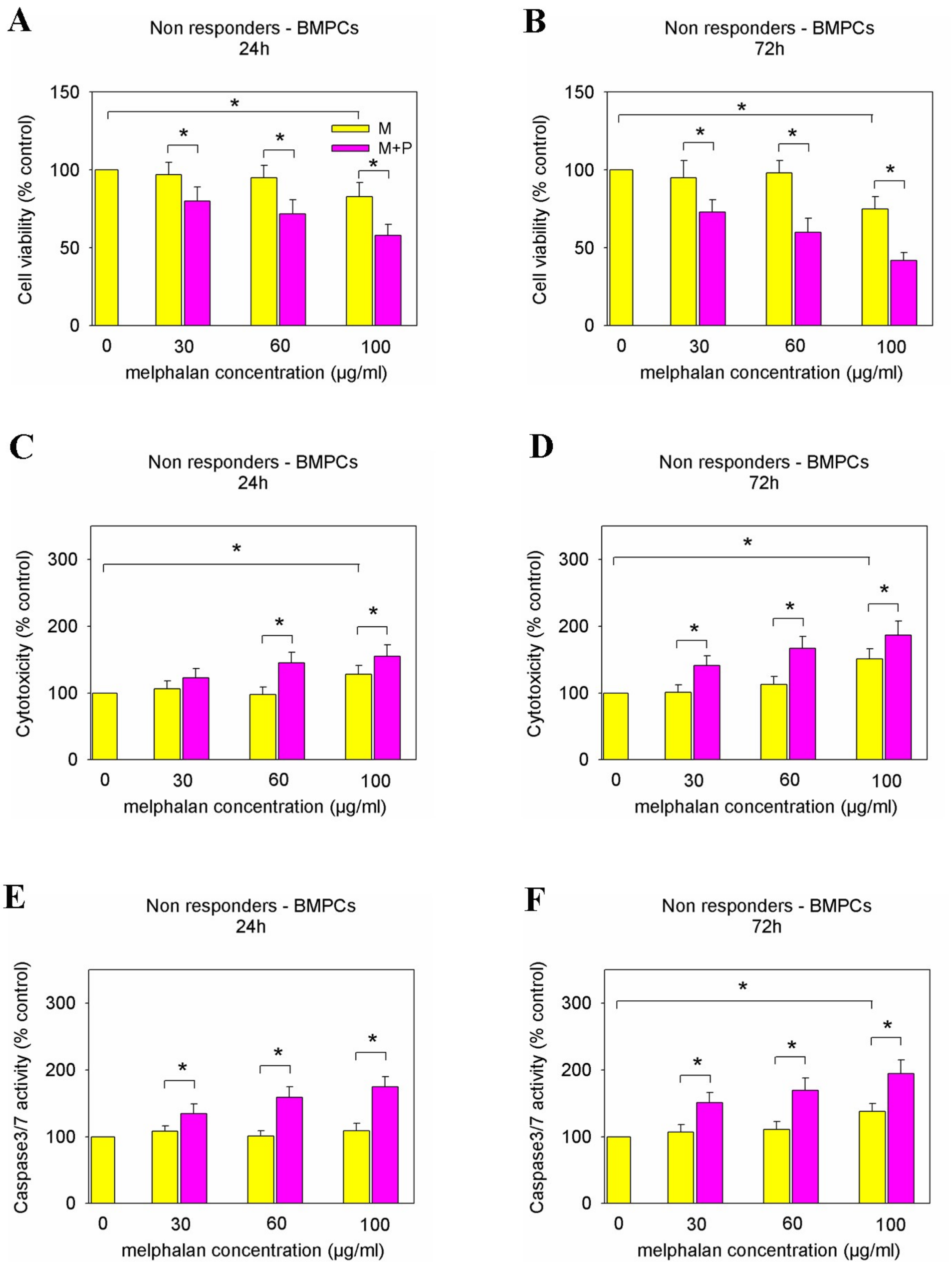

Figure S4. ApoTox-Glo triplex assay of BMPCs from non-responders patients. The viability (**A,B**), cytotoxicity (**C,D**), and apoptosis (**E,F**) of BMPCs from non-responders 24h (**A,C,E**) and 72h (**B,D,F**) after treatment with various doses of melphalan (0-100µg/ml) in combination or not of 5nM of panobinostat. Data are presented as the mean ± SD; *$p < 0.05$.

A

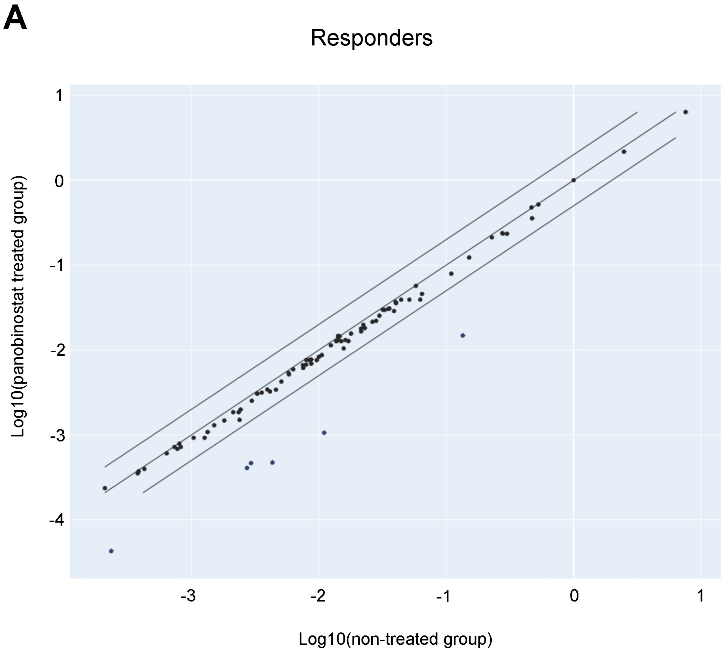

B

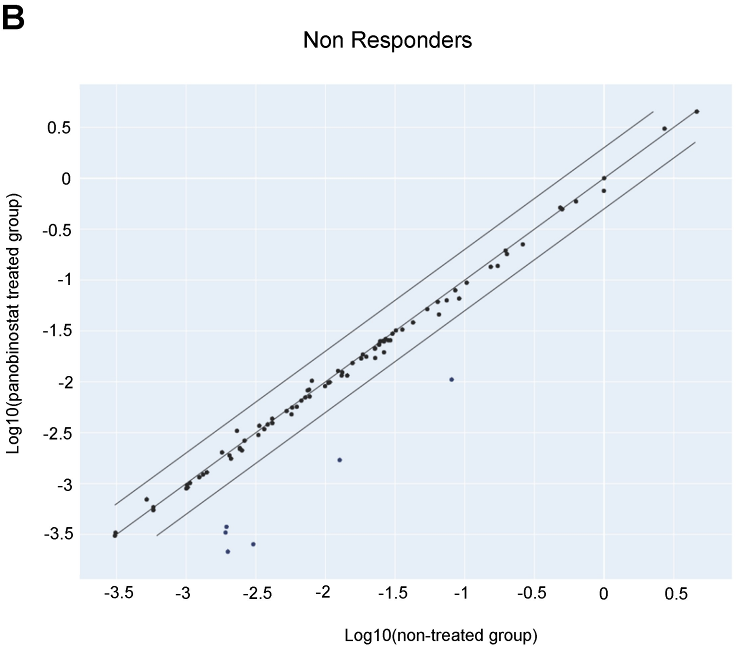

Figure S5. Significant gene expression changes between panobinostat treated and non-treated BMPCs - Scatter Plot. BMPCs from responders (**A**) and non-responders (**B**) patients were analyzed. The center diagonal line indicates unchanged gene expression, while the outer diagonal lines indicate the selected fold regulation threshold (Fold Regulation Threshold = 2). Genes with data points beyond the outer lines in the upper left and lower right corners are up-regulated or down-regulated, respectively, by more than the fold regulation threshold in the y-axis Group relative to the x-axis Group.

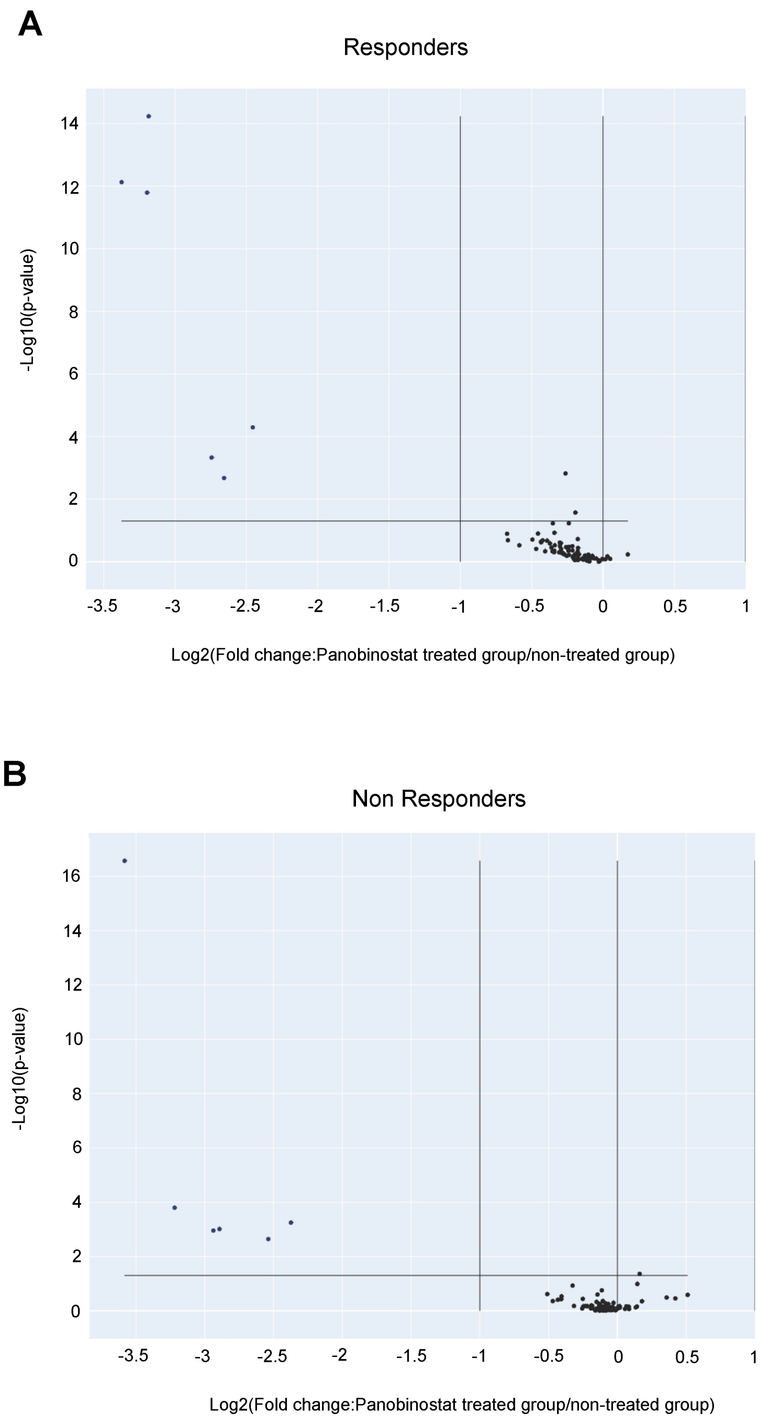

Figure S6. Significant gene expression changes between panobinostat treated and non-treated BMPCs - Volcano Plot. BMPCs from responders (**A**) and non-responders (**B**) patients were analyzed. The center vertical line indicates unchanged gene expression, while the two outer vertical lines indicate the selected fold regulation threshold (*p*-Value Threshold = 0.05). The horizontal line indicates the selected *p*-value threshold. Genes with data points in the far upper left (down-regulated) and far upper right (up-regulated) sections meet the selected fold regulation and *p*-value thresholds.

A Responders

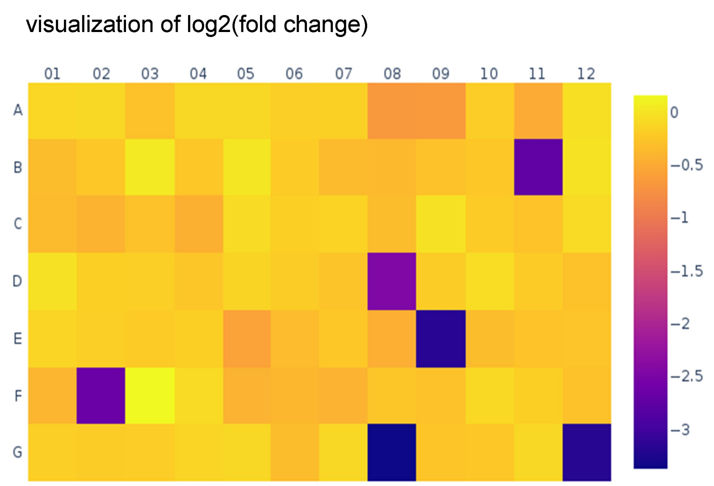

Layout	01	02	03	04	05	06	07	08	09	10	11	12
A	ABL1 / -1.09	APEX1 / -1.08	ATM / -1.24	ATR / -1.08	ATRIP / -1.08	ATRX / -1.13	BARD1 / -1.12	BAX / -1.59	BBC3 / -1.59	BLM / -1.14	BRCA1 / -1.41	BRIP1 / -1.03
B	CDC25A / -1.26	CDC25C / -1.18	CDK7 / 1.04	CDKN1A / -1.19	CHEK1 / 1.01	CHEK2 / -1.16	CIB1 / -1.28	CRY1 / -1.29	CSNK2A2 / -1.23	DDB1 / -1.18	DDB2 / -6.7	DDIT3 / -1.02
C	ERCC1 / -1.28	ERCC2 / -1.34	EXO1 / -1.23	FANCA / -1.37	FANCD2 / -1.05	FANCG / -1.13	FEN1 / -1.11	GADD45A / -1.27	GADD45G / -1.02	H2AFX / -1.16	HUS1 / -1.22	LIG1 / -1.06
D	MAPK12 / -1.02	MBD4 / -1.13	MCPH1 / -1.13	MDC1 / -1.19	MLH1 / -1.1	MLH3 / -1.15	MPG / -1.21	MRE11A / -5.48	MSH2 / -1.15	MSH3 / -1.04	NBN / -1.16	NTHL1 / -1.23
E	OGG1 / -1.09	PARP1 / -1.13	PCNA / -1.16	PMS1 / -1.13	PMS2 / -1.5	PNKP / -1.28	PPM1D / -1.18	PPP1R15A / -1.38	PRKDC / -9.1	RAD1 / -1.27	RAD17 / -1.21	RAD18 / -1.21
F	RAD21 / -1.32	RAD50 / -6.31	RAD51 / 1.13 / B	RAD51B / -1.06	RAD9A / -1.35	RBBP8 / -1.31	REV1 / -1.35	RNF168 / -1.2	RNF8 / -1.23	RPA1 / -1.07	SIRT1 / -1.13	SMC1A / -1.23
G	SUMO1 / -1.13	TOPBP1 / -1.15	TP53 / -1.15	TP53BP1 / -1.09	TP73 / -1.08	UNG / -1.27	XPA / -1.08	XPC / -10.37	XRCC1 / -1.2	XRCC2 / -1.18	XRCC3 / -1.07	XRCC6 / -9.17

B Non Responders

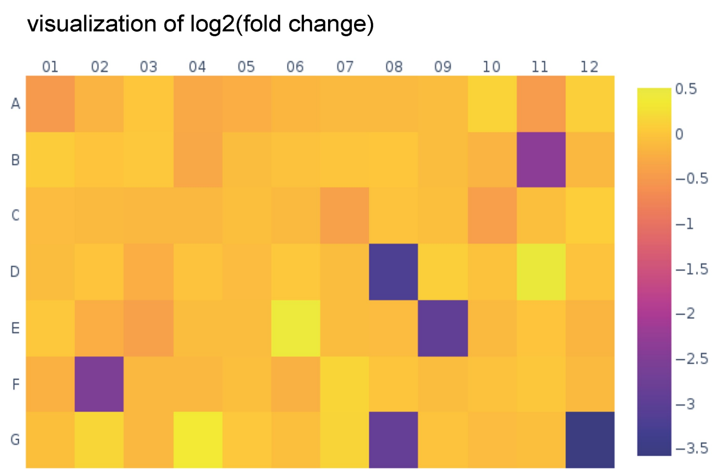

Layout	01	02	03	04	05	06	07	08	09	10	11	12
A	ABL1 / -1.42	APEX1 / -1.14	ATM / -1.0	ATR / -1.24	ATRIP / -1.19	ATRX / -1.12	BARD1 / -1.09	BAX / -1.09	BBC3 / -1.06	BLM / 1.1	BRCA1 / -1.39	BRIP1 / 1.06
B	CDC25A / 1.04	CDC25C / -1.02	CDK7 / 1.01	CDKN1A / -1.25	CHEK1 / -1.07	CHEK2 / -1.04	CIB1 / -1.01	CRY1 / -1.0	CSNK2A2 / -1.08	DDB1 / -1.14	DDB2 / -5.18	DDIT3 / -1.11
C	ERCC1 / -1.09	ERCC2 / -1.1	EXO1 / -1.12	FANCA / -1.11	FANCD2 / -1.05	FANCG / -1.1	FEN1 / -1.33	GADD45A / -1.03	GADD45G / -1.05	H2AFX / -1.35	HUS1 / -1.06	LIG1 / 1.05
D	MAPK12 / -1.08	MBD4 / -1.02	MCPH1 / -1.19	MDC1 / -1.03	MLH1 / -1.08	MLH3 / 1.01	MPG / -1.07	MRE11A / -9.31 / A	MSH2 / 1.06	MSH3 / -1.03	NBN / 1.42	NTHL1 / -1.03
E	OGG1 / 1.01	PARP1 / -1.19	PCNA / -1.33	PMS1 / -1.08	PMS2 / -1.07	PNKP / 1.34	PPM1D / -1.07	PPP1R15A / -1.08	PRKDC / -7.66	RAD1 / -1.1	RAD17 / -1.02	RAD18 / -1.13
F	RAD21 / -1.16	RAD50 / -5.81	RAD51 / -1.12	RAD51B / -1.11	RAD9A / -1.06	RBBP8 / -1.17	REV1 / 1.1	RNF168 / -1.01	RNF8 / -1.07	RPA1 / -1.04	SIRT1 / -1.0	SMC1A / -1.1
G	SUMO1 / -1.05	TOPBP1 / 1.12	TP53 / -1.11	TP53BP1 / 1.28	TP73 / 1.01	UNG / -1.05	XPA / 1.11	XPC / -7.43	XRCC1 / -1.03	XRCC2 / -1.08	XRCC3 / -1.06	XRCC6 / -11.98 / A

Figure S7. Heat Map - BMPCs. Test group: Responders (**A**) or non-responders (**B**) MM patients, BMPCs *ex vivo* treated with panobinostat. Control Group: Non-treated Responders (**A**) or non-responders (**B**) MM patients, untreated BMPCs.

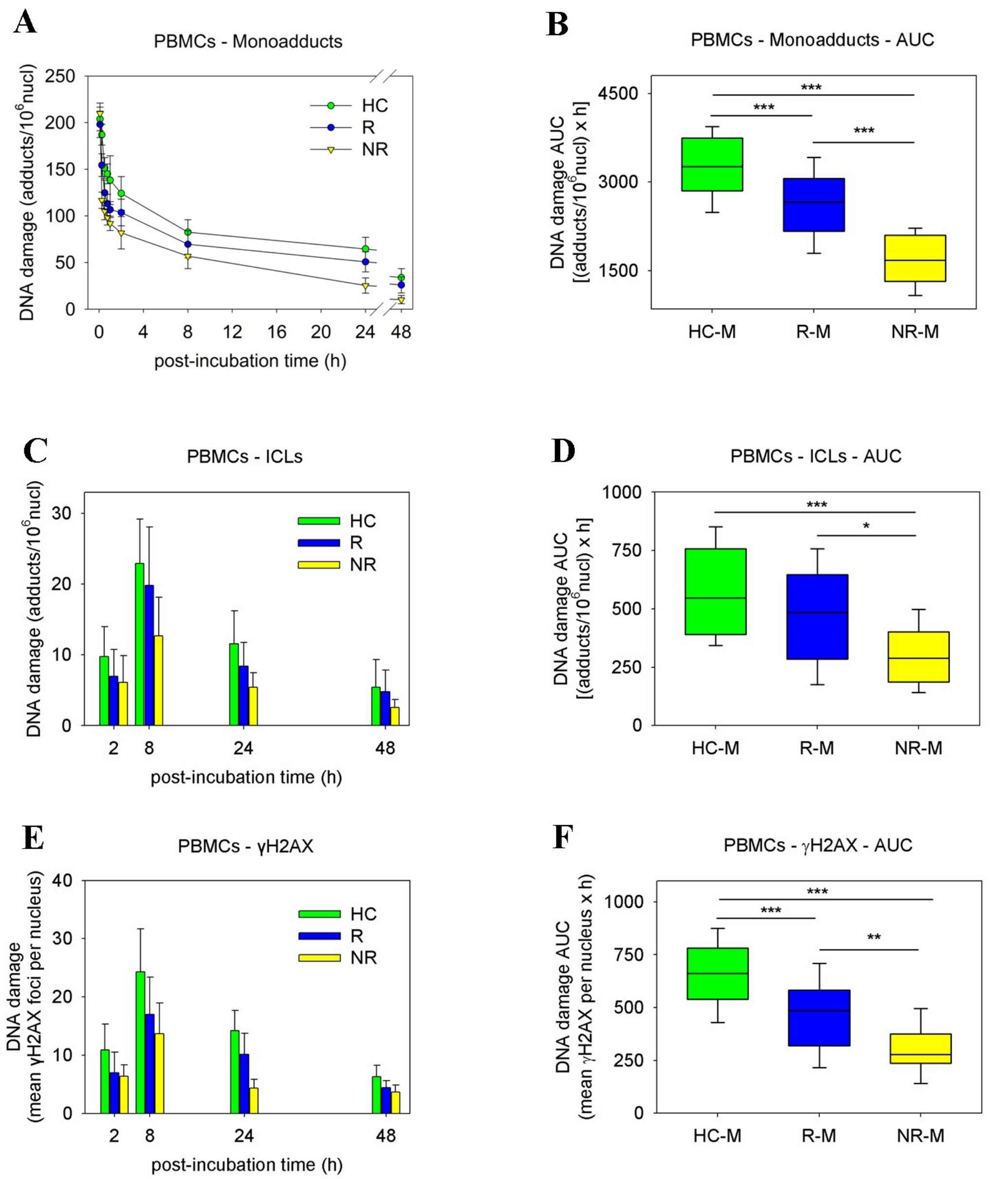

Figure S8. Kinetics of melphalan-induced DNA damage formation and repair in PBMCs. (**A**) The kinetics of monoadducts repair 0-48h after treatment of PBMCs from healthy controls (HC) and MM patients, responders (R) or non-responders (NR), with melphalan. (**B**) Accumulation of DNA damage (expressed as AUC) following treatment with melphalan. The formation and repair of ICLs (**C**) and the accumulation of ICLs (**D**) after treatment with melphalan. The formation and removal of γH2AX foci (**E**) and the accumulation of γH2AX foci (**F**) after treatment with melphalan. The experiments shown were based on a minimum of three independent repeats; *$p < 0.05$, **$p < 0.01$, ***$p < 0.001$.

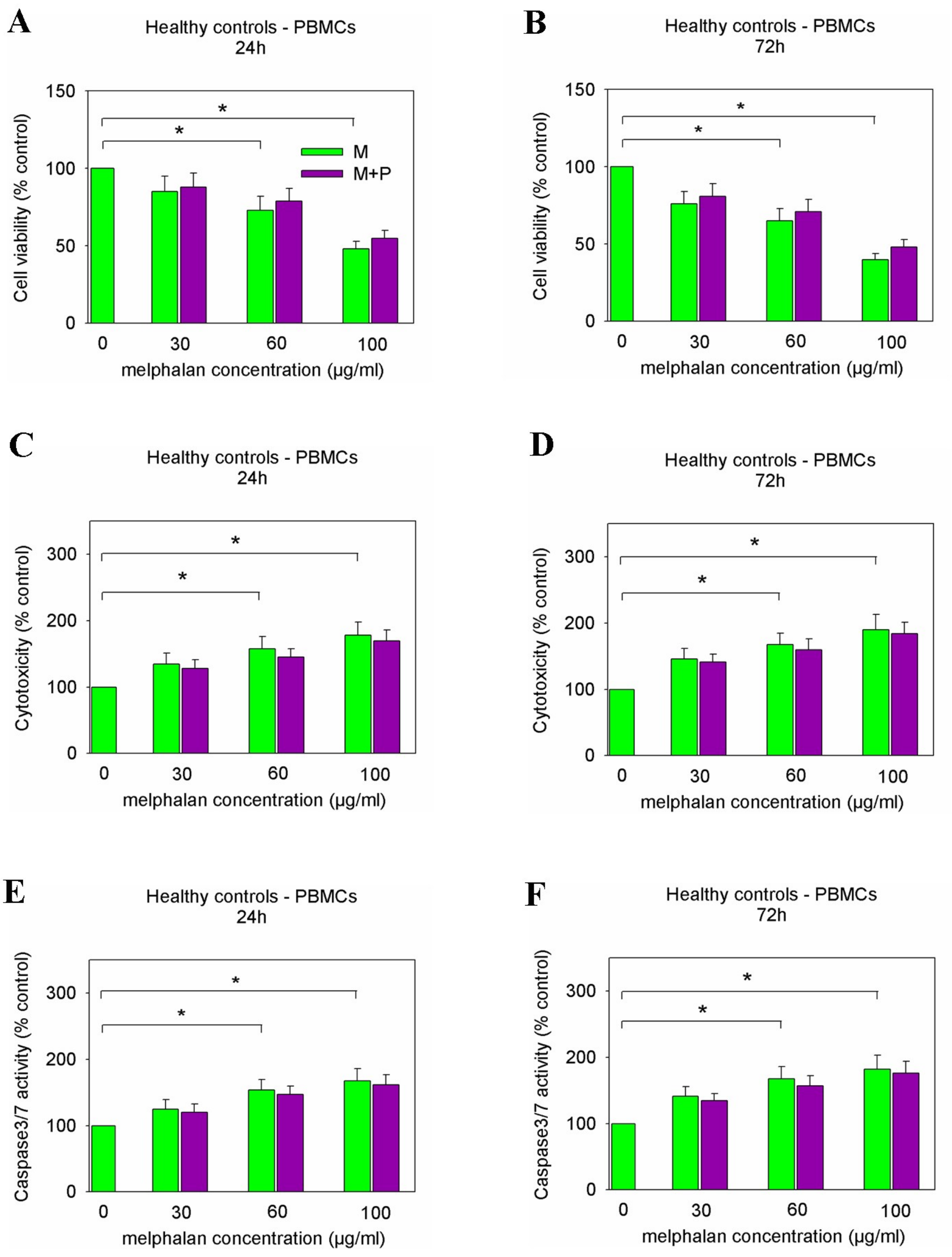

Figure S9. ApoTox-Glo triplex assay of PBMCs from healthy controls. The viability (**A,B**), cytotoxicity (**C,D**), and apoptosis (**E,F**) of PBMCs from healthy controls 24h (**A,C,E**) and 72h (**B,D,F**) after treatment with various doses of melphalan (0-100µg/ml) in combination or not of 5nM of panobinostat. Data are presented as the mean ± SD; *$p < 0.05$.

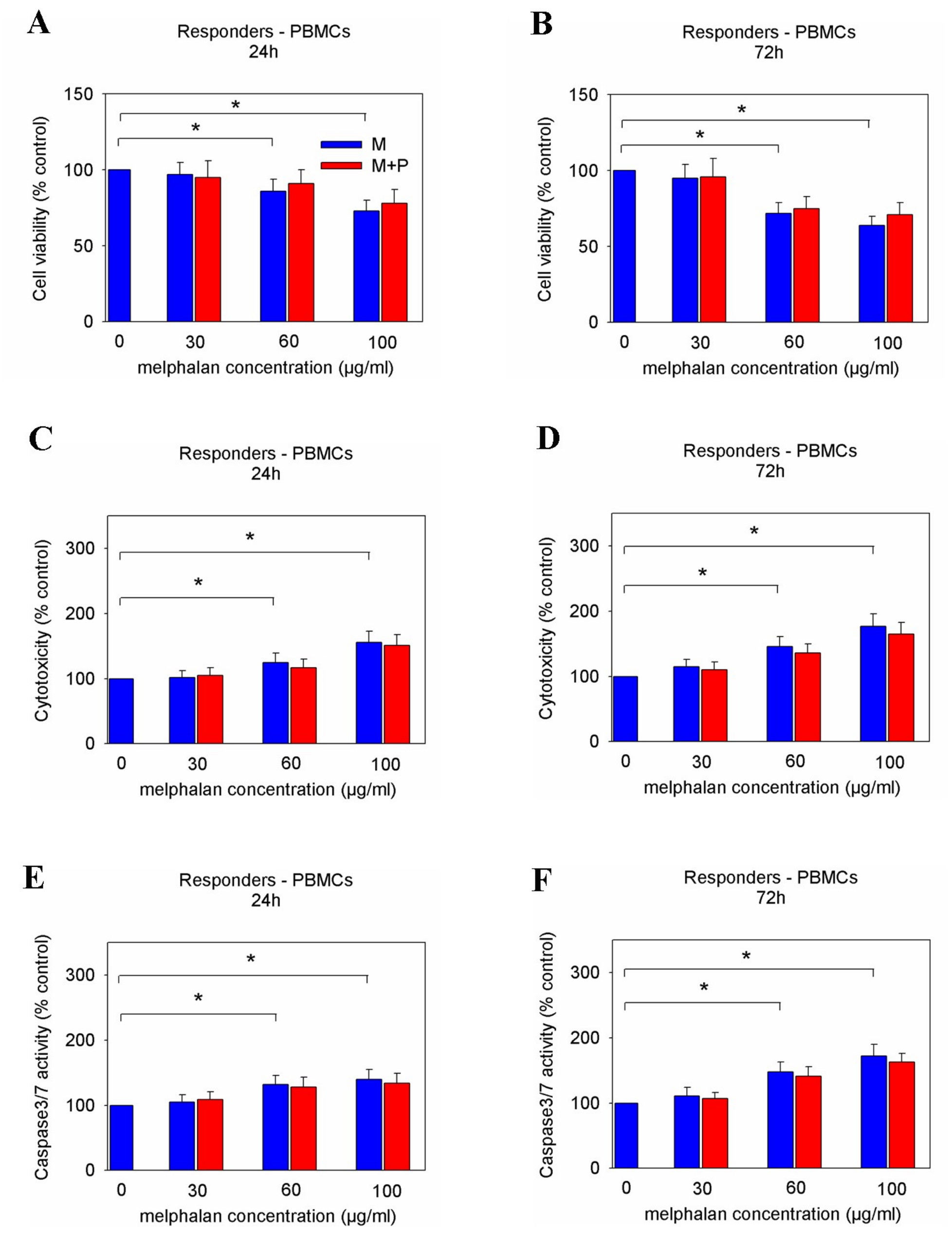

Figure S10. ApoTox-Glo triplex assay of PBMCs from responders patients. The viability (A,B), cytotoxicity (**C,D**), and apoptosis (**E,F**) of PBMCs from responders 24h (**A,C,E**) and 72h (**B,D,F**) after treatment with various doses of melphalan (0-100µg/ml) in combination or not of 5nM of panobinostat. Data are presented as the mean ± SD; **p* < 0.05.

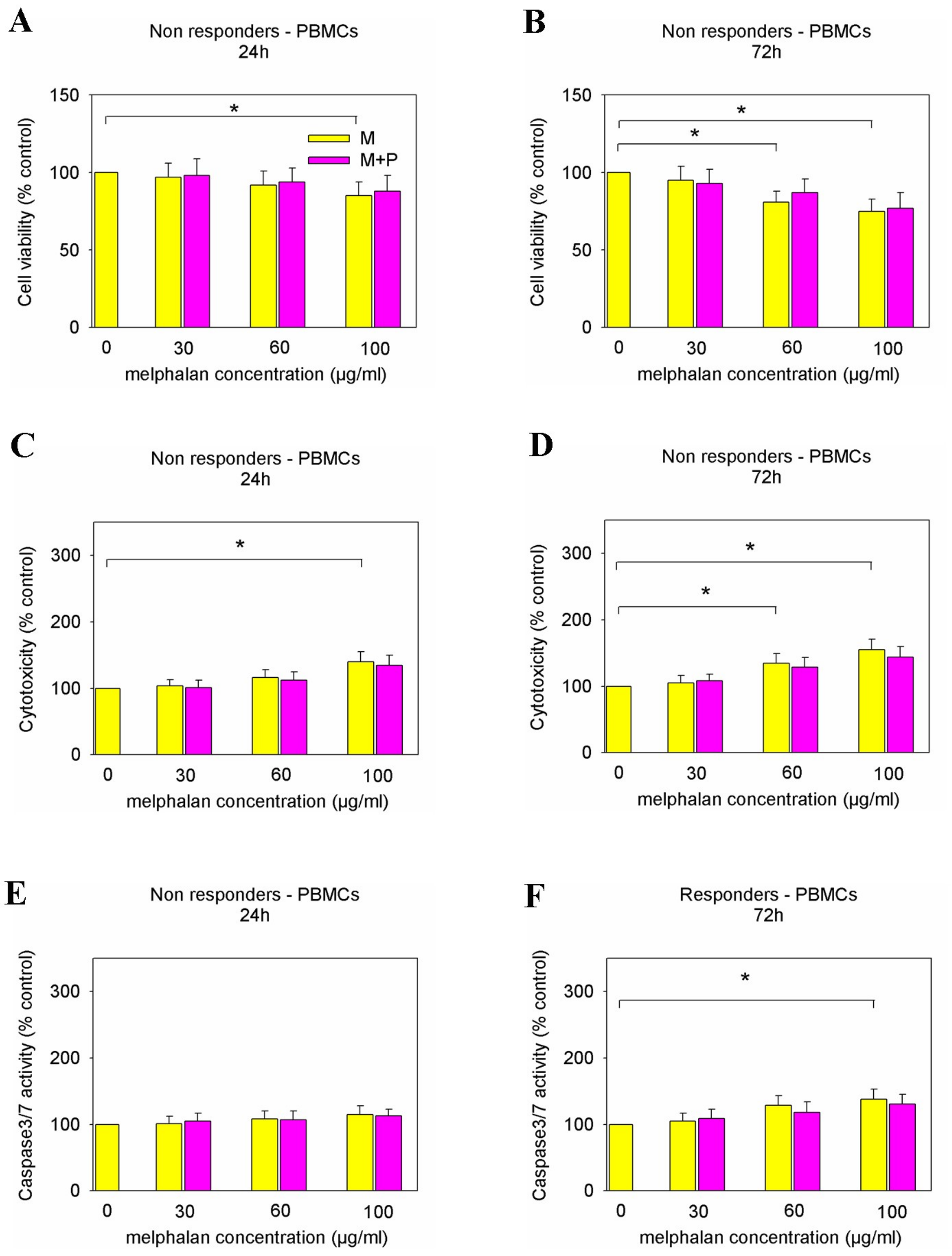

Figure S11. ApoTox-Glo triplex assay of PBMCs from non-responders patients. The viability (A,**B**), cytotoxicity (**C,D**), and apoptosis (**E,F**) of PBMCs from non-responders 24h (**A,C,E**) and 72h (**B,D,F**) after treatment with various doses of melphalan (0-100µg/ml) in combination or not of 5nM of panobinostat. Data are presented as the mean ± SD; *$p < 0.05$.

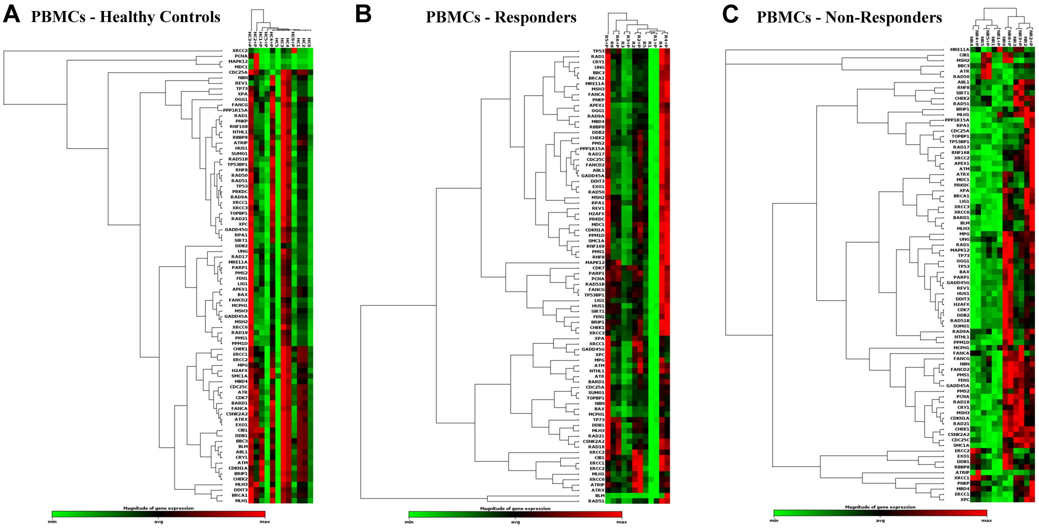

Figure S12. Panobinostat treatment on the expression of DDR-associated genes in PBMCs - Hierarchical clustergrams. Critical DDR-associated genes were analyzed in PBMCs from (**A**) 6 healthy controls, (**B**) 6 responders and (**C**) 6 non-responders MM patients. Test group: PBMCs *ex vivo* treated with panobinostat. Control Group: non-treated PBMCs from the same subjects.

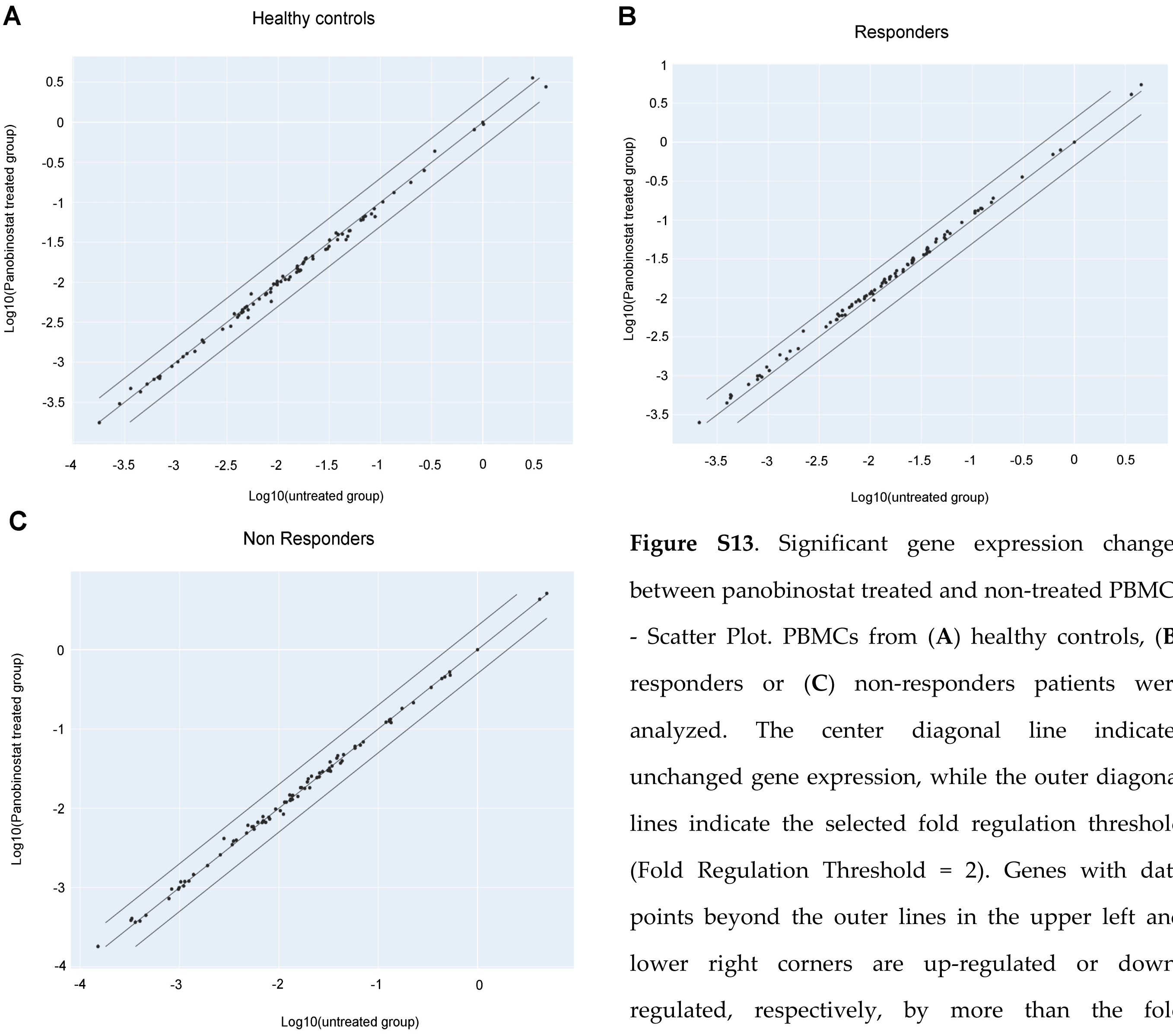

Figure S13. Significant gene expression changes between panobinostat treated and non-treated PBMCs - Scatter Plot. PBMCs from (**A**) healthy controls, (**B**) responders or (**C**) non-responders patients were analyzed. The center diagonal line indicates unchanged gene expression, while the outer diagonal lines indicate the selected fold regulation threshold (Fold Regulation Threshold = 2). Genes with data points beyond the outer lines in the upper left and lower right corners are up-regulated or down-regulated, respectively, by more than the fold regulation threshold in the y-axis Group relative to the x-axis Group.

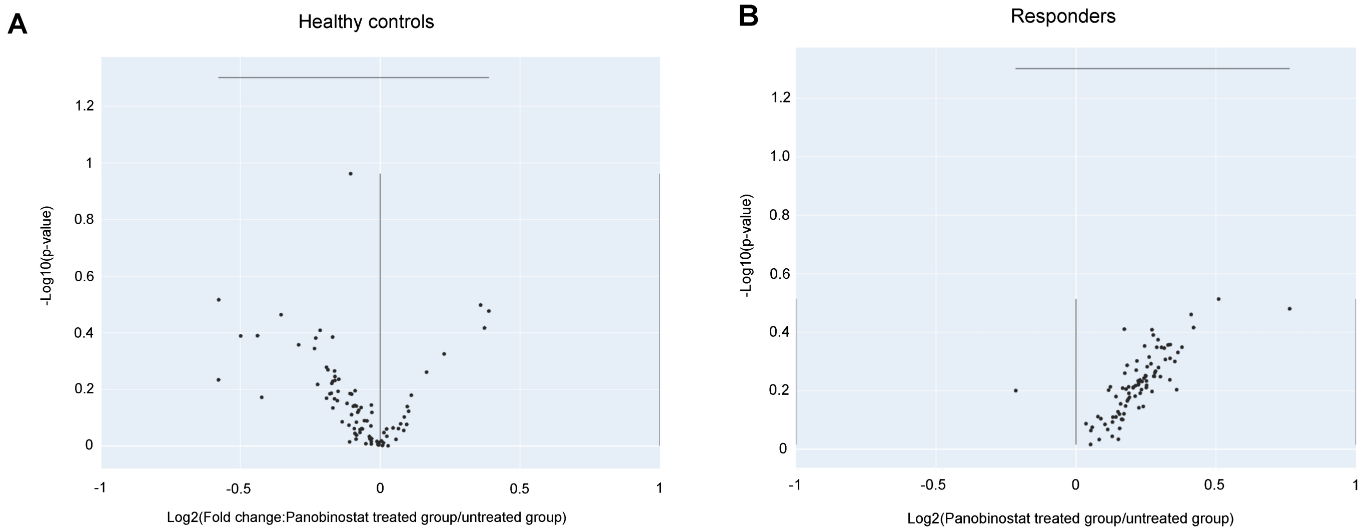

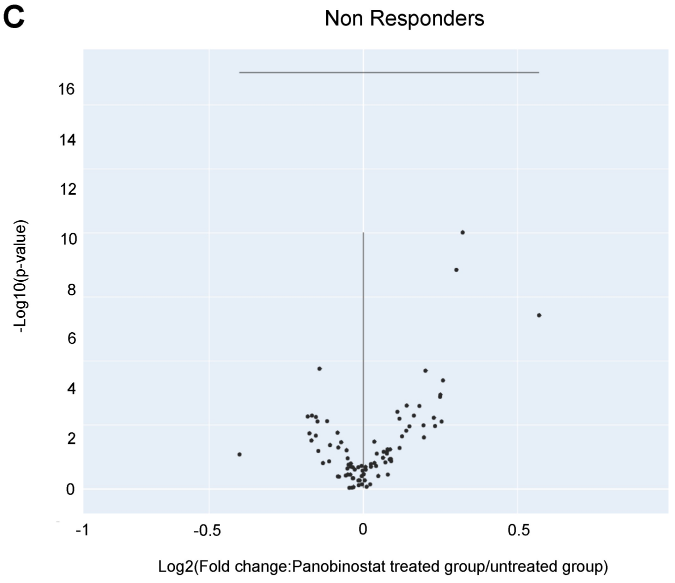

Figure S14. Significant gene expression changes between panobinostat treated and non-treated PBMCs - Volcano Plot. PBMCs from (**A**) healthy controls, (**B**) responders or (**C**) non-responders patients were analyzed. The center vertical line indicates unchanged gene expression, while the two outer vertical lines indicate the selected fold regulation threshold. The horizontal line indicates the selected *p*-value threshold (*p*-Value Threshold = 0.05). Genes with data points in the far upper left (down-regulated) and far upper right (up-regulated) sections meet the selected fold regulation and *p*-value thresholds.

A Healthy controls

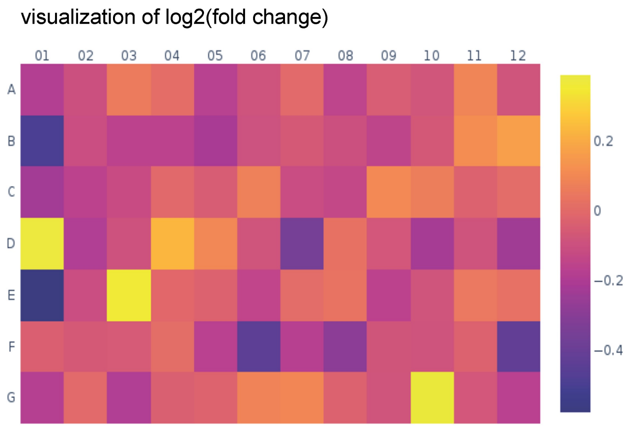

Layout	01	02	03	04	05	06	07	08	09	10	11	12
A	ABL1 / -1.14	APEX1 / -1.07	ATM / 1.04	ATR / 1.01	ATRIP / -1.13	ATRX / -1.06	BARD1 / -1.0	BAX / -1.11	BBC3 / -1.03	BLM / -1.05	BRCA1 / 1.06	BRIP1 / -1.06
B	CDC25A / -1.41	CDC25C / -1.08	CDK7 / -1.12	CDKN1A / -1.12	CHEK1 / -1.16	CHEK2 / -1.07	CIB1 / -1.05	CRY1 / -1.07	CSNK2A2 / -1.11	DDB1 / -1.05	DDB2 / 1.08	DDIT3 / 1.12
C	ERCC1 / -1.17	ERCC2 / -1.12	EXO1 / -1.09	FANCA / -1.01	FANCD2 / -1.04	FANCG / 1.05	FEN1 / -1.08	GADD45A / -1.1	GADD45G / 1.07	H2AFX / 1.05	HUS1 / -1.02	LIG1 / 1.0
D	MAPK12 / 1.29	MBD4 / -1.14	MCPH1 / -1.07	MDC1 / 1.17	MLH1 / 1.07	MLH3 / -1.06	MPG / -1.28	MRE11A / 1.02	MSH2 / -1.05	MSH3 / -1.17	NBN / -1.06	NTHL1 / -1.18
E	OGG1 / -1.49	PARP1 / -1.08	PCNA / 1.28	PMS1 / -1.01	PMS2 / -1.02	PNKP / -1.11	PPM1D / 1.01	PPP1R15A / 1.02	PRKDC / -1.12	RAD1 / -1.06	RAD17 / 1.03	RAD18 / 1.02
F	RAD21 / -1.03	RAD50 / -1.05	RAD51 / -1.04	RAD51B / 1.01	RAD9A / -1.12	RBBP8 / -1.36	REV1 / -1.13	RNF168 / -1.22	RNF8 / -1.06	RPA1 / -1.06	SIRT1 / -1.02	SMC1A / -1.34
G	SUMO1 / -1.13	TOPBP1 / -1.0	TP53 / -1.14	TP53BP1 / -1.03	TP73 / -1.02 / B	UNG / 1.06	XPA / 1.07	XPC / -1.02	XRCC1 / -1.06	XRCC2 / 1.31	XRCC3 / -1.05	XRCC6 / -1.12

B Responders

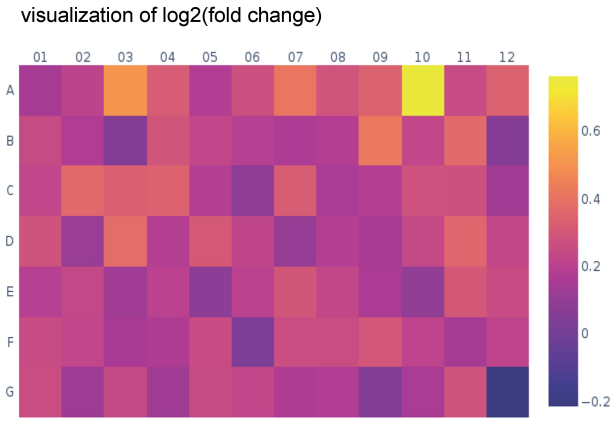

Layout	01	02	03	04	05	06	07	08	09	10	11	12
A	ABL1 / 1.1	APEX1 / 1.16	ATM / 1.42	ATR / 1.24	ATRIP / 1.13	ATRX / 1.2	BARD1 / 1.33	BAX / 1.22	BBC3 / 1.26	BLM / 1.7	BRCA1 / 1.19	BRIP1 / 1.26
B	CDC25A / 1.19	CDC25C / 1.13	CDK7 / 1.04	CDKN1A / 1.22	CHEK1 / 1.17	CHEK2 / 1.14	CIB1 / 1.12	CRY1 / 1.14	CSNK2A2 / 1.34	DDB1 / 1.17	DDB2 / 1.29	DDIT3 / 1.04
C	ERCC1 / 1.17	ERCC2 / 1.28	EXO1 / 1.26	FANCA / 1.26	FANCD2 / 1.14	FANCG / 1.06	FEN1 / 1.25	GADD45A / 1.11	GADD45G / 1.13	H2AFX / 1.21	HUS1 / 1.21	LIG1 / 1.09
D	MAPK12 / 1.21	MBD4 / 1.08	MCPH1 / 1.3	MDC1 / 1.13	MLH1 / 1.24	MLH3 / 1.16	MPG / 1.07	MRE11A / 1.14	MSH2 / 1.11	MSH3 / 1.18	NBN / 1.28	NTHL1 / 1.17
E	OGG1 / 1.14	PARP1 / 1.18	PCNA / 1.1	PMS1 / 1.15	PMS2 / 1.06	PNKP / 1.15	PPM1D / 1.23	PPP1R15A / 1.17	PRKDC / 1.12	RAD1 / 1.06	RAD17 / 1.23	RAD18 / 1.19
F	RAD21 / 1.19	RAD50 / 1.17	RAD51 / 1.11	RAD51B / 1.12	RAD9A / 1.19	RBBP8 / 1.03	REV1 / 1.2	RNF168 / 1.19	RNF8 / 1.23	RPA1 / 1.16	SIRT1 / 1.1	SMC1A / 1.16
G	SUMO1 / 1.19	TOPBP1 / 1.09	TP53 / 1.18	TP53BP1 / 1.09	TP73 / 1.18 / B	UNG / 1.17	XPA / 1.12	XPC / 1.13	XRCC1 / 1.04	XRCC2 / 1.11	XRCC3 / 1.22	XRCC6 / -1.16

C Non Responders

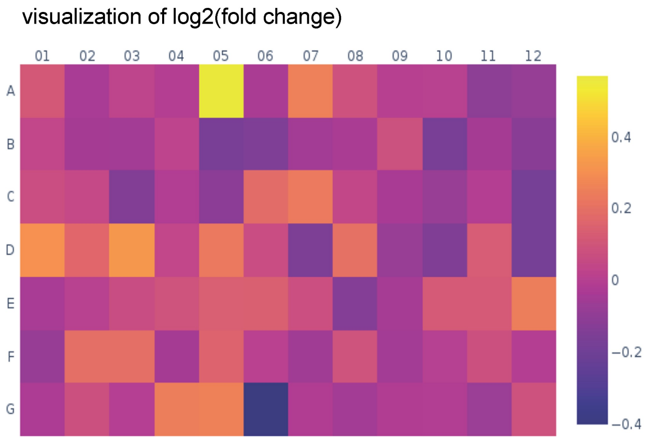

Layout	01	02	03	04	05	06	07	08	09	10	11	12
A	ABL1 / 1.08	APEX1 / -1.02	ATM / 1.02	ATR / -1.0	ATRIP / 1.48	ATRX / -1.02	BARD1 / 1.19	BAX / 1.06	BBC3 / 1.0	BLM / 1.0	BRCA1 / -1.08	BRIP1 / -1.06
B	CDC25A / 1.02	CDC25C / -1.03	CDK7 / -1.04	CDKN1A / 1.02	CHEK1 / -1.12	CHEK2 / -1.11	CIB1 / -1.04	CRY1 / -1.02	CSNK2A2 / 1.06	DDB1 / -1.12	DDB2 / -1.03	DDIT3 / -1.08
C	ERCC1 / 1.05	ERCC2 / 1.03	EXO1 / -1.1	FANCA / -1.01	FANCD2 / -1.08	FANCG / 1.13	FEN1 / 1.17	GADD45A / 1.03	GADD45G / -1.03	H2AFX / -1.06	HUS1 / -1.0	LIG1 / -1.13
D	MAPK12 / 1.23	MBD4 / 1.12	MCPH1 / 1.25	MDC1 / 1.03	MLH1 / 1.17	MLH3 / 1.05	MPG / -1.11	MRE11A / 1.15	MSH2 / -1.06	MSH3 / -1.11	NBN / 1.09	NTHL1 / -1.13
E	OGG1 / -1.02	PARP1 / 1.01	PCNA / 1.04	PMS1 / 1.06	PMS2 / 1.1	PNKP / 1.1	PPM1D / 1.05	PPP1R15A / -1.1	PRKDC / -1.03	RAD1 / 1.08	RAD17 / 1.08	RAD18 / 1.19
F	RAD21 / -1.06	RAD50 / 1.14	RAD51 / 1.15	RAD51B / -1.03	RAD9A / 1.11	RBBP8 / 1.01	REV1 / -1.04	RNF168 / 1.06	RNF8 / -1.04	RPA1 / 1.0	SIRT1 / 1.05	SMC1A / -1.0
G	SUMO1 / -1.02	TOPBP1 / 1.06	TP53 / -1.0	TP53BP1 / 1.19	TP73 / 1.2 / B	UNG / -1.32	XPA / -1.01	XPC / -1.04	XRCC1 / -1.01	XRCC2 / -1.01	XRCC3 / -1.05	XRCC6 / 1.06

Figure S15. Heat Map - PBMCs. Test group: (**A**) Healthy controls, (**B**) responders or (**C**) non-responders MM patients, PBMCs *ex vivo* treated with panobinostat. Control Group: (**A**) Healthy controls, (**B**) responders or (**C**) non-responders MM patients, untreated PBMCs.
